# A Practical Review of Proteasome Pharmacology

**DOI:** 10.1124/pr.117.015370

**Published:** 2019-04

**Authors:** Tiffany A. Thibaudeau, David M. Smith

**Affiliations:** Department of Biochemistry, West Virginia University School of Medicine, Morgantown, West Virginia

## Abstract

The ubiquitin proteasome system (UPS) degrades individual proteins in a highly regulated fashion and is responsible for the degradation of misfolded, damaged, or unneeded cellular proteins. During the past 20 years, investigators have established a critical role for the UPS in essentially every cellular process, including cell cycle progression, transcriptional regulation, genome integrity, apoptosis, immune responses, and neuronal plasticity. At the center of the UPS is the proteasome, a large and complex molecular machine containing a multicatalytic protease complex. When the efficiency of this proteostasis system is perturbed, misfolded and damaged protein aggregates can accumulate to toxic levels and cause neuronal dysfunction, which may underlie many neurodegenerative diseases. In addition, many cancers rely on robust proteasome activity for degrading tumor suppressors and cell cycle checkpoint inhibitors necessary for rapid cell division. Thus, proteasome inhibitors have proven clinically useful to treat some types of cancer, especially multiple myeloma. Numerous cellular processes rely on finely tuned proteasome function, making it a crucial target for future therapeutic intervention in many diseases, including neurodegenerative diseases, cystic fibrosis, atherosclerosis, autoimmune diseases, diabetes, and cancer. In this review, we discuss the structure and function of the proteasome, the mechanisms of action of different proteasome inhibitors, various techniques to evaluate proteasome function in vitro and in vivo, proteasome inhibitors in preclinical and clinical development, and the feasibility for pharmacological activation of the proteasome to potentially treat neurodegenerative disease.

## I. Introduction

Early biologists viewed cellular proteins as essentially stable constituents subjected to only minor “wear and tear.” The widely accepted theory was that dietary proteins functioned primarily as energy, providing fuel for the body. Rudolf Schoenheimer and colleagues challenged that notion in the late 1930s, using stable isotopes to show that trace dietary amino acids rapidly incorporated into tissue proteins (Schoenheimer et al., 1939) and that these proteins are in a dynamic state of synthesis and degradation (Schoenheimer, 1942). Today we understand that all intracellular proteins are continually “turning over” (i.e., they are being hydrolyzed into their constituent amino acids and replaced via de novo synthesis). Individual proteins are degraded at different rates, varying from minutes for certain regulatory enzymes to days for myosin heavy chains in cardiac muscle and to months for hemoglobin in erythrocytes (Lecker et al., 2006). Although cytosolic proteins can be degraded in lysosomes (via chaperone-mediated autophagy and macroautophagy), the majority are degraded by the proteasome (Glickman and Ciechanover, 2002).

The discovery of a special class of cytoplasmic granules containing acid hydrolases in the 1950s (de Duve et al., 1953; Appelmans et al., 1955), called lysosomes (de Duve et al., 1955), was an important step forward in understanding intracellular protein breakdown. Breakdown of endogenous (autophagy) and exogenous (heterophagy) material was believed to occur in lysosomes, the “intracellular digestive system” (de Duve and Wattiaux, 1966). Because peptide hydrolysis is an exergonic (i.e., downhill) reaction, the discovery of ATP-dependent protein breakdown in mammalian (Simpson, 1953) cells was unexpected. Simpson (1953) suggested “the possible existence of two (or more) mechanisms of protein breakdown, one hydrolytic, the other energy-requiring.” Subsequent work over the next 2 decades firmly established that both rates of protein synthesis and degradation determine the cellular protein concentration as well as the wide variability of protein half-lives (Schimke, 1964; Schimke and Doyle, 1970; Zavortink et al., 1979).

Studies in the 1970s supported the prediction of a new selective degradation pathway that accounted for the wide distribution of protein half-lives (Goldberg, 1972; Goldberg and Dice, 1974; Poole et al., 1976). Interestingly, cytosolic proteins synthesized with structural analogs of normal amino acids are rapidly degraded within the cell (Goldberg, 1972; Knowles and Ballard, 1976). These seminal observations added another layer of selectivity in which the inherent stability of each protein also determines the degradation rate, presumably to prevent the accumulation of abnormal proteins. However, the mechanism of selectivity remained a mystery. ATP was found to be essential for protein catabolism, but it was unknown whether a proteolytic step was directly dependent on ATP or whether it required some additional reactions (Goldberg and St John, 1976). Selective and ATP-dependent protein degradation was not congruent with the notion of the lysosome as the key player in protein breakdown. What could be responsible for this exquisitely controlled protein degradation? Etlinger and Goldberg (1977) identified a novel, soluble, ATP-dependent proteolytic system that was independent from the lysosome. The importance of the soluble degradation system was emphasized when Rechsteiner and colleagues showed that most intracellular proteins are degraded in the cytosol, not the lysosome (Bigelow et al., 1981).

Wilk and Orlowski (1980) purified a 700-kDa “multicatalytic proteinase complex” (later shown to be the 20S proteasome). Unlike all other known proteases, this new protease complex could cleave peptides after basic, acidic, or hydrophobic residues, suggesting that it contained multiple distinct active sites (Wilk and Orlowski, 1980, 1983). Electron micrographs revealed the complex to be a 700-kDa stacked “donut” ring structure (Tanaka et al., 1988). Due to their critical roles in intracellular protein breakdown, these protease complexes were collectively renamed “proteasomes” (Arrigo et al., 1988). Analogous protease complexes of equivalent size, shape, polypeptide composition, and proteolytic activities have since been identified across all three domains of life (Tanaka et al., 1988; Goldberg, 2007).

The next major advancement in the field came with the discovery of ubiquitin, a small approximately 8-kDa protein with a big role in protein degradation. Aaron Ciechanover and colleagues identified a small heat-stable protein, ubiquitin, covalently conjugated to target substrates (Ciechanover et al., 1978) in an ATP-dependent manner (Ciechanover et al., 1980; Wilkinson et al., 1980). This led to the proposed model in which protein-substrate modification by several ubiquitin moieties targets it for degradation by a downstream, as-yet-unidentified protease that cannot recognize the unmodified substrate (Hershko et al., 1980). It was later shown that some nonubiquitin proteins are also degraded in an ATP-dependent manner (Tanaka et al., 1983). Rechsteiner’s group later went on to purify the ATP-dependent 26S proteasome responsible for ubiquitin conjugate degradation (Hough et al., 1986, 1987).

**Fig. 1.**
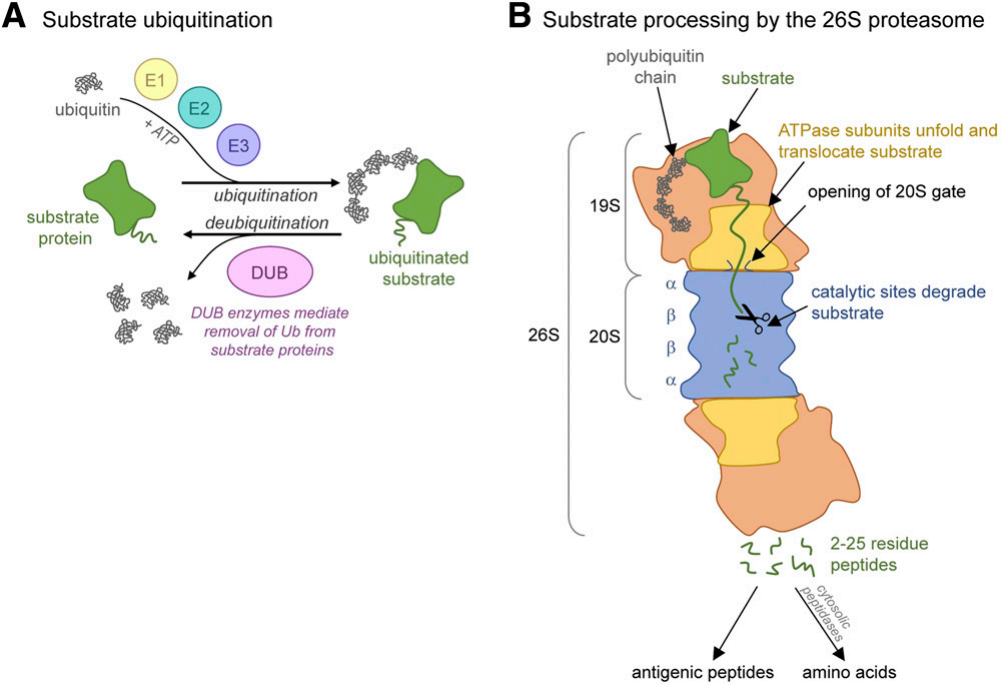
The ubiquitin proteasome pathway. (A) Simplified model of the ubiquitin conjugation system. (B) Primary steps involved in ubiquitinated substrate processing by the 26S proteasome.

Avram Hershko, Aaron Ciechanover, and Irwin Rose characterized the system of ubiquitin conjugation and its role in marking proteins for degradation (Hershko et al., 1983), an achievement that earned them the Nobel Prize in Chemistry (2004) (Ciechanover, 2005). Attachment of poly-ubiquitin chains to specific proteins selects them for proteasome-mediated degradation (Fig. 1A). Targeting proteins for degradation requires three enzymatic components to link chains of ubiquitin onto selected protein substrates. E1 (ubiquitin-activating enzyme) and E2s (ubiquitin-carrier or conjugating proteins) prepare ubiquitin for conjugation. The E3 (ubiquitin-protein ligase) enzymes control substrate specificity, recognizing substrate degradation signals and catalyzing the transfer of activated ubiquitin to the substrate (Ciechanover et al., 1982; Hershko et al., 1983). Eukaryotic cells contain hundreds of E3 ligases, allowing the cell to precisely control ubiquitination and degradation of individual proteins (Ciechanover, 2013). Ubiquitin conjugation is necessary for cell viability (Ciechanover et al., 1984; Finley et al., 1984) and activity of the ubiquitin pathway is greatly increased in cells making abnormal proteins (Hershko et al., 1982).

During the past 20 years, investigators have established the critical role of the ubiquitin proteasome system (UPS) in cell cycle progression (Hershko and Ciechanover, 1998; Peters, 2002; Bassermann et al., 2014), transcriptional regulation, genome integrity, apoptosis, immune responses (Ben-Neriah, 2002), and the pathogenesis of many human diseases (Glickman and Ciechanover, 2002; Sakamoto, 2002). With respect to disease, the proteasome is particularly important for maintaining cellular protein homeostasis (i.e., proteostasis). When the efficiency of proteostasis systems declines, misfolded and damaged proteins aggregate to toxic levels within the cell, potentially giving rise to many neurodegenerative diseases (Layfield et al., 2005; McKinnon and Tabrizi, 2014). Too much of a good thing can be just as detrimental. Cancer cells rely on robust proteasome activity for degrading tumor suppressors and cell cycle checkpoint inhibitors necessary for rapid cell division (Dou et al., 2003). Numerous processes rely on finely tuned proteasome function, making it a crucial target for therapeutic intervention in many diseases, including neurodegenerative diseases, cystic fibrosis, atherosclerosis, autoimmune diseases, diabetes, and cancer (Schmidt and Finley, 2014). In 2003, bortezomib (Velcade; Takeda Pharmaceuticals, Cambridge, MA) became the first U.S. Food and Drug Administration (FDA)–approved proteasome inhibitor as a third-line treatment of multiple myeloma (MM).

In this review, we first discuss the structure, function, and regulation of the proteasome. Then we discuss the classes and mechanisms of action of proteasome inhibitors. Next, we summarize commonly used in vitro and in vivo techniques for studying proteasome activity and inhibition, followed by a review of currently FDA-approved proteasome inhibitors as well as novel inhibitors undergoing clinical and preclinical trials. Finally, we discuss how pharmacological activation of the proteasome could produce novel therapeutics to treat neurodegenerative disease.

## II. Proteasome Structure and Function

### A. Proteasome Structure and Activity

The 26S proteasome is a 2.4-MDa molecular machine that makes up nearly 2% of total cellular protein (Kisselev and Goldberg, 2001). It is composed of a 20S proteasome core particle capped on one or both ends by the 19S regulatory particle (Fig. 1B). It degrades proteins by a multistep process; the 19S regulatory particle binds ubiquitinated substrates, opens a substrate entry gate in 20S (DeMartino et al., 1996; Adams et al., 1998a), and unfolds its substrates by linearly translocating them into the 20S catalytic chamber, where they are degraded to peptides (DeMartino and Slaughter, 1999; Voges et al., 1999). Numerous studies over the past 2 decades have developed our present understanding of proteasome structure and function. The first 20S core particle was crystalized in the late 1990s (Groll et al., 1997). Since then, hundreds of 20S structures, complexed with regulators or inhibitors, have been solved.

Eukaryotic proteasomes contain four stacked heteroheptameric rings arranged in a *α*_7_-*β*_7_-*β*_7_-*α*_7_ fashion (Groll et al., 1997). The amino (N) termini of the *α* subunits form a “gate,” folding over the 13-Å central pore and occluding access to the proteolytic sites located on the *β*-subunit lumen (Groll et al., 2000). Passage through this gate is the rate-limiting step and prevents unregulated protein degradation (Köhler et al., 2001). The *α*N-terminal tails are highly conserved, containing a tyrosine-aspartate-arginine (YDR) motif that forms salt bridges with neighboring tails that obstruct the 13-Å entry pore. The *α*3 N terminus is the lynchpin, critical for stabilizing the closed-gate confirmation (Köhler et al., 2001). Note that the purified latent 20S proteasome still exhibits a degree of peptidase activity due to stochastic conformational fluctuations within the N termini (Religa et al., 2010; Ruschak and Kay, 2012). Interestingly, deletion of the first eight *α*3 residues (*α*3∆N-20S) sufficiently destabilizes the closed-gate conformation and accelerates the entry and degradation of peptides (Köhler et al., 2001). *α*3∆N-20S crystallographic structures show that the remaining N termini are disordered, resulting in a constitutively open gate (Köhler et al., 2001). Wild-type 20S proteasome activity is similarly accelerated when bound to a proteasome activator (e.g., 19S/PA700, 11S/PA28, Blm10/PA200) (Finley et al., 2016). Proteasome activators bind to a free end of the *α*-subunit ring and “open” the gate by distinct mechanisms that are discussed in detail below.

#### *1. Active Sites of the 20S Proteasome*

**Fig. 2.**
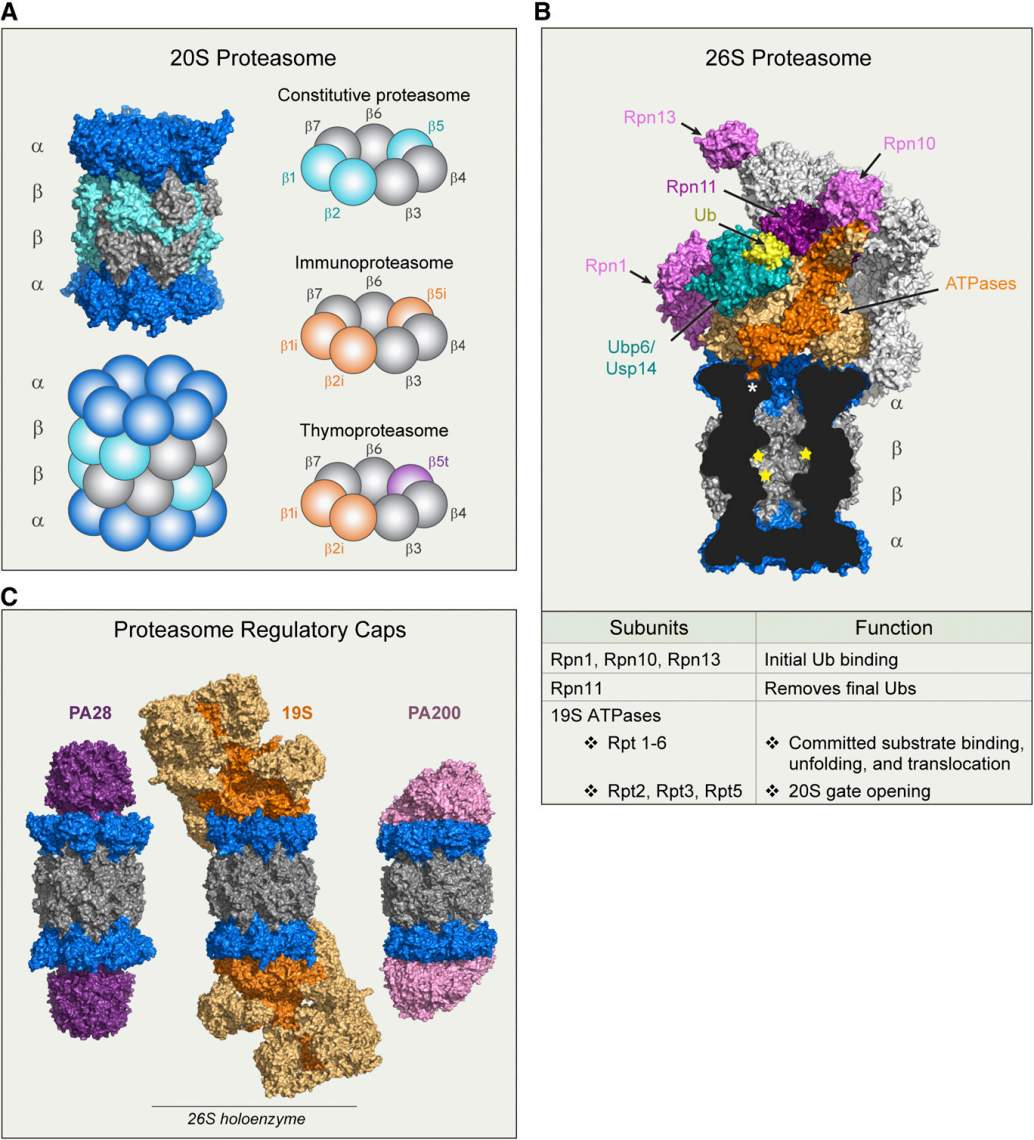
Proteasome structure and function. (A) Structures (PDB 4r3o) and cartoon representation of 20S proteasome, highlighting the different *β*-subunit combinations found in tissue-specific proteasomes discussed in the text. (B) Structure of the 26S proteasome in complex with Ubp6 (PDB 5a5b). A cross-section of 20S proteasome reveals the C terminus of Rpt5 ATPase (dark orange) positioned in the inter-*α*-subunit pocket (asterisk). Proteolytic sites are marked with yellow stars. Labeled 19S subunits are discussed in the text. (C) 20S proteasomes (blue and gray) complexed with regulatory caps: PA28 homolog PA26 (PDB 1fnt), 19S (PDB 5gjr), and PA200 yeast homolog Blm10 (PDB 4v7o). 19S ATPases are dark orange, and non-ATPase subunits are light orange. PDB, Protein Data Bank.

The eukaryotic 20S proteasome contains six proteolytically active *β* subunits, three on each *β* ring, that exhibit different substrate preferences (Fig. 2, A and B). The various substrate binding pockets determine active site specificity like classic proteases but with more complexities. The binding pockets themselves are formed by specific interactions between the catalytic subunit and the neighboring *β* subunit (Borissenko and Groll, 2007). As a result, the proteasome is not simply a complex of independent proteases but is a unique multicatalytic enzyme functioning only when wholly intact. The chymotrypsin-like site (*β*5) preferentially cleaves after hydrophobic residues, the trypsin-like site (*β*2) preferentially cleaves after basic residues, and the caspase-like site (*β*1) preferentially cleaves after acidic residues (Arendt and Hochstrasser, 1997; Voges et al., 1999). Despite their names, these sites do not share the catalytic mechanisms of their namesakes and their substrate preferences are much broader than the names imply (Kisselev and Goldberg, 2001). Multiple catalytic sites with varying specificities advantageously allow for the rapid and processive degradation of cellular proteins.

All proteasome active sites use an N-terminal threonine nucleophile. Enzyme inhibitor and site-directed mutagenesis studies compose much of what we know about the proteasome’s unusual catalytic mechanism (Groll and Huber, 2004). Although proteasomes lack the classic catalytic triad found in cysteine and serine proteases, the proteasome’s sensitivity to peptide aldehyde inhibitors suggests a similar catalytic mechanism (Groll and Huber, 2004). Accordingly, the crystal structure of 20S bound to the peptide aldehyde Ac-Leu-Leu-nLeu-al (ALLN) reveals a hemiacetyl bond between the *β*-subunit N-terminal threonine hydroxyl groups (Groll and Huber, 2004). Proteasome inhibitors (lactacystin, vinyl sulfones, and epoxyketones) are often found to covalently modify this threonine. As expected, mutation to a serine residue retains significant activity, whereas mutation to an alanine residue completely abolishes activity (Groll and Huber, 2004).

#### *2. Specialized Catalytic Subunits*

Some cell types express *β* subunits with modified catalytic sites under certain conditions. Immune cells constitutively express alternative catalytic subunits (*β*1i/LMP2, *β*2i/MECL-1, and *β*5i/LMP7) that are preferentially incorporated into the 20S proteasome de novo in place of the constitutive *β*1 (*β*1c), *β*2 (*β*2c), and *β*5 (*β*5c) subunits, forming the immunoproteasome (Tanaka, 1994) (Fig. 2A). The immunoproteasome is also expressed in nonhematopoietic cells when exposed to interferon (IFN)-*γ* or tumor necrosis factor-*α* (Tanaka, 1994). To our knowledge the main purpose of the immunoproteasome is to enhance ligand generation for major histocompatibility complex class I (MHC-I) molecules (Rock and Goldberg, 1999) that allow for immune surveillance (Van Kaer et al., 1994). How do these immune subunits do this? These subunits use the same catalytic mechanism as their constitutively expressed counterparts but they have different cleavage preferences due to changes in substrate binding pockets (Gaczynska et al., 1993; Groll and Huber, 2004). The most striking difference between constitutive and immunoproteasomes is the *β*1i subunit, which lacks caspase-like activity but instead cleaves after hydrophobic residues (Groll and Huber, 2004). This is a crucial difference because only MHC-I molecules with tightly bound ligands are expressed on the cell surface. Tight class I binding requires ligands eight to nine amino acids in length with either a hydrophobic or basic anchor residue at the C terminus. Ligands with acidic C termini are not accepted (Falk and Rötzschke, 1993; Kniepert and Groettrup, 2014). Thus, the immunoproteasome facilitates the production of peptides suitable for MHC-I presentation (Rock and Goldberg, 1999).

Human thymus cortical epithelial cells express a thymic-specific catalytic subunit, *β*5t; *β*5t, *β*1i, and *β*2i together form the thymoproteasome (Murata et al., 2007) (Fig. 1A). The thymoproteasome is essential for T-lymphocyte positive selection. Compared with *β*5c and *β*5i, *β*5t has weak chymotrypsin-like activity; thus, it is speculated that thymoproteasomes facilitate the low-affinity MHC-I molecule ligand production necessary for positive selection (Murata et al., 2007). Further details on unique functions of tissue-specific proteasomes can be found in Kniepert and Groettrup (2014).

#### *3. Proteasome Regulatory Caps and Their Diverse Biologic Roles*

Regulation of gate opening in the 20S proteasome is an important aspect of proteasome function; as such, the cell has evolved many different proteasomal regulators that control 20S gate opening (Finley et al., 2016). The most well known regulator is 19S (PA700), a component of the 26S proteasome. The 26S proteasome is a structurally dynamic complex, adopting large-scale conformational changes around the central axis during the ATP-dependent processing of substrates (Beck et al., 2012; Matyskiela et al., 2013; Śledź et al., 2013; Bashore et al., 2015). These structural changes appear to be necessary for substrate protein unfolding and injection into the 20S core particle.

Ubiquitin-dependent degradation requires several steps: 1) substrate binding and commitment, 2) 20S gate opening, 3) substrate unfolding and translocation, and 4) deubiquitination (Fig. 2B). First, the 19S regulatory particle has three integral subunits that serve as substrate receptors: Rpn1, Rpn10, and Rpn13. These substrate receptors reversibly associate with ubiquitin, and have only low affinity for mono-ubiquitin (de Poot et al., 2017). The multiplicity of ubiquitin receptors coupled with a variety of shuttling factors, such as proteins that have a ubiquitin-like (UBL) domain and a ubiquitin-associating domain, allows the 26S proteasome to recognize and degrade many types of ubiquitin conjugates (Collins and Goldberg, 2017). Substrate binding to the ubiquitin receptors induces a conformational change aligning the 19S ATPase translocation channel directly over the 20S gate (Bashore et al., 2015), induces gate opening, and stimulates ATP hydrolysis (Peth et al., 2009, 2013). Substrate commitment requires a loosely folded region of the protein (e.g., unstructured initiation site) to insert into the ATPase ring in an ATP-dependent manner (Peth et al., 2010). Tyrosine pore loops inside the ATPases “grip” the substrate and this tight association enables the processive process of substrate unfolding and translocation into the 20S core (Collins and Goldberg, 2017; Snoberger et al., 2017). Second, the six ATPase subunits (Rpt1–6) form a ring at the bottom of the 19S complex with their C termini inserting into the 20S *α*-subunit intersubunit pockets (Rabl et al., 2008). 19S-dependent gate opening requires ATPase C-terminal hydrophobic-tyrosine-any residue (HbYX) motif binding to intersubunit pockets (between the *α* subunits) on top of the 20S (Yu et al., 2010). Binding of the 19S C termini to the 20S is thought to induce a conformational change in the *α* subunits, opening the gate (Rabl et al., 2008). The exact mechanisms behind the 26S HbYX-motif gate opening in human 26S proteasomes are not clear. However, binding of an HbYX-motif peptide (the last eight residues of Rpt5) is sufficient to allosterically induce conformational changes in the 20S *α* subunit and open the gate (Smith et al., 2007). Third, the six ATPase subunits power processive unfolding and translocation of substrates into the 20S core coupled with ATP hydrolysis (Smith et al., 2006; Beckwith et al., 2013). Fourth, Rpn11 is the integral proteasome-associated deubiquitinase (DUB) enzyme of the 19S complex. Rpn11 is positioned directly above the translocation channel when substrate is committed for degradation and removes the entire ubiquitin chain as proteins are translocated (de Poot et al., 2017). Two other DUBs are transiently associated with proteasomes (Usp14 and Uch37) and they can trim substrate ubiquitin chains prior to the committed step, rescuing the substrate from degradation (de Poot et al., 2017). Proteasome-associated DUBs are discussed in section VI.

In addition to 19S, there are two other proteasome gate activator families, 11S and PA200/Blm10, neither of which contains unfoldase activity or requires ATP (Fig. 2C). Higher eukaryotes express three 11S regulatory subunits: PA28*α*,*β*,*γ* (also known as REG*α*,*β*,*γ*) (Johnston et al., 1997; Rechsteiner and Hill, 2005). PA28*αβ* forms an inducible heteroheptamer that is primarily located in the cytoplasm. In contrast, PA28*γ* forms a homoheptamer that is constitutively expressed in the nucleus (Baldin et al., 2008; Finley et al., 2016). The biologic roles of these regulators remain relatively mysterious. However, both forms of PA28 regulators show increased expression after acute oxidative stress in cells, suggesting that both play a significant role in oxidized protein degradation (Pickering and Davies, 2012). In addition, IFN-*γ* increases PA28*αβ* expression, oxidized protein degradation capacity (Pickering and Davies, 2012), and MHC-I ligand generation.

PA200 (Blm10 in yeast) plays a role in spermatogenesis (Khor et al., 2006), response to DNA repair (Ustrell et al., 2002), glutamine homeostasis (Blickwedehl et al., 2012), and mitochondrial inheritance (Sadre-Bazzaz et al., 2010), although molecular details behind many of these functions are not clear. The crystal structure of yeast Blm10-20S shows that Blm10 forms a large HEAT repeat-like solenoid in a 1.5 superhelical turn, forming a dome that caps the ends of the 20S proteasome (Sadre-Bazzaz et al., 2010). One C-terminal HbYX motif binds between the *α*5- and *α*6-intersubunit pocket and facilitates gate opening (Ortega et al., 2005; Schmidt et al., 2005). As with the 19S Rpt5 subunit, an eight-amino-acid Blm10 C-terminal fragment (Blm-pep) induces gate opening in purified 20S proteasomes (Witkowska et al., 2017). The Blm-pep–bound 20S crystal structure closely resembles the bound HbYX in the full-length Blm10 structure. PA200/Blm10-containing proteasomes specifically catalyze the acetylation-dependent, but not polyubiquitination-dependent, core histone degradation during somatic DNA damage response and spermatogenesis (Qian et al., 2013). During spermatogenesis, the spermatoproteasome (formed by PA200, 20S, *β*1i, *β*2i, *β*5i, and the human testis-specific *α*4_s_) degrades core histones and is required for proper sperm maturation and viability (Qian et al., 2013). The existence of interchangeable proteasome subunits highlights the cell’s repertoire of proteasome complexes tailored for specific cellular roles.

### B. Proteasome-Dependent Cellular Processes

**Fig. 3.**
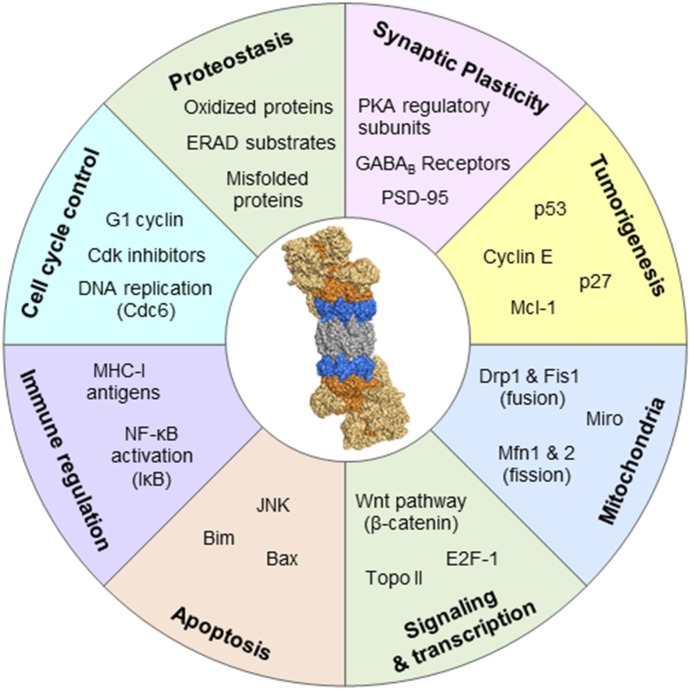
Examples of cellular functions that depend on proteasome function. Important pathways dependent on proteasome function and exemplar substrates. Bax, bcl-2-like protein 4; Bim, bcl-2-like protein 11; Cdk, cyclin-dependent kinase; Drp1, dynamin-1-like protein; ERAD, endoplasmic-reticulum-associated protein degradation; E2F-1, target of retinoblastoma protein; Fis1, mitochondrial fission 1 protein; GABA, gamma-aminobutyric acid; JNK, C-Jun-amino-terminal kinase; Mfn, mitofusin; MHC-I, major histocompatibility complex-I; Miro, mitochondrial Rho GTPase; NF-*κ*B, nuclear factor–*κ*B; PKA, protein kinase A; PSD-95, postsynaptic density protein 95; Topo II, type II topoisomerase; Wnt, wingless-type.

Proteasome function is essential to cellular homeostasis. In addition to maintaining proteostasis, the proteasome plays a key role in regulating many physiologic processes. Four major areas not previously discussed include cell cycle regulation, nuclear factor-*κ*B (NF-*κ*B) activation, neuronal function, and endoplasmic reticulum (ER)–associated protein degradation (Fig. 3).

#### 1. Cell Cycle

The proteasome degrades many cell cycle regulatory proteins that typically have short half-lives (e.g., cyclin B1, p21, p27) and tumor suppressors (e.g., p53) promoting cycle progression (Dietrich et al., 1996; Machiels et al., 1997; Adams et al., 1999; Wu et al., 2000; Shah et al., 2001). Not surprisingly, most cancers heavily rely on proteasome activity and are more susceptible to proteasome inhibition than normal cells (Dulić et al., 1994; Pagano et al., 1995; King et al., 1996). Proteasome inhibition in cancer is discussed in section IV.

#### 2. Nuclear Factor-κB Activation

The NF-*κ*B transcription factors (NF-*κ*B and Rel proteins) regulate expression of genes involved in innate and adaptive immunity, inflammation, stress responses, B-cell development, and lymphoid organogenesis. In cancer cells, NF-*κ*B is critically involved in the expression of the antiapoptotic IAP family of genes as well as BCL-2 prosurvival genes (Wang et al., 1998; Zong et al., 1999; Chen et al., 2000).

Canonical and noncanonical NF-*κ*B activation requires proteasome-mediated degradation of regulatory elements for transcriptional activation. In the unstimulated state, the inhibitory I*κ*B subunits bind and sequester NF-*κ*B/Rel complexes in the cytoplasm (Baldwin, 2001). In canonical pathway activation, proinflammmatory cytokines activate the I*κ*B kinase (IKK) complex (IKK*β*, IKK*α*, and NF-*κ*B essential modulator), which phosphorylates I*κ*B, leading to I*κ*B ubiquitination and proteasomal degradation (Chen et al., 1995; Scherer et al., 1995; Spencer et al., 1999; Winston et al., 1999), freeing NF-*κ*B/RelA complexes. Freed NF-*κ*B/RelA complexes translocate to the nucleus, where they (either alone in or combination with other transcription factors) induce target gene expression. In the noncanonical pathway, NF-*κ*B–p100/RelB complexes are inactive in the cytoplasm. Signaling activates the NF-*κ*B–inducing kinase, which activates IKK*α* complexes that phosphorylate NF-*κ*B2–p100 C-terminal residues. Phosphorylated NF*κ*B is ubiquitinated and processed by the proteasome into NF-*κ*B2–p52, which is transcriptionally competent. Such processing by the proteasome is remarkable since it requires the initiation of protein degradation, followed by termination of degradation at a specific domain, demonstrating exquisite control over degradation. After processing, NF-*κ*B2–p52 translocates to the nucleus and induces target gene expression. NF-*κ*B is a prosurvival pathway and is upregulated in many cancers and inflammatory diseases (Wang et al., 1996). Given the indispensable role of proteasome function in activating this pathway, proteasome inhibition is a valid therapeutic target. The role of NF-*κ*B in cancer pathology is discussed further in section V.

#### 3. Neuronal Function

Maintaining proteostasis in neurons is especially important due to their complex architecture, long lifespan, and inability to dilute aggregate load through cell division (Tai and Schuman, 2008). Importantly, the UPS is critical for normal functioning of neuronal synapses, including synaptic protein turnover, plasticity, and long-term memory formation, which rely on tightly controlled changes in the proteome (Fonseca et al., 2006; Tai and Schuman, 2008; Aso et al., 2012; Djakovic et al., 2012). In addition to the intracellular proteasomes, Ramachandran and Margolis (2017) identified a mammalian nervous system–specific membrane-associated proteasome complex that rapidly modulates neuronal function. This proteasome complex degrades intracellular proteins and releases the products into the synaptic cleft, where they stimulate postsynaptic *N*-methyl-d-aspartate receptor–dependent neuronal signaling, a process important for regulating synaptic function.

The accumulation of aggregation-prone proteins is a hallmark of neurodegenerative disease commonly accompanied by loss of proteostasis and progressive death of neurons (Selkoe, 2003; Rubinsztein, 2006; Brettschneider et al., 2015). It is established that proteasome function is decreased in neurodegenerative diseases, and its impairment is implicated in the development of many neurodegenerative diseases, including Alzheimer, Parkinson, and Huntington diseases (Keller et al., 2000; McNaught et al., 2001; Ciechanover and Brundin, 2003; Rubinsztein, 2006; Ortega et al., 2007). To highlight this point, brain region–specific proteasome inhibition closely mirrors the neuropathology and clinical hallmarks of neurodegenerative diseases (McNaught et al., 2002, 2004; Bedford et al., 2008; Li et al., 2010). The importance of targeting the proteasome for potential neurodegenerative disease therapy is discussed in section VI.

#### 4. Endoplasmic Reticulum–Associated Protein Degradation and the Unfolded Protein Response

Secretory proteins and most integral membrane proteins are synthesized and enter the ER lumen for proper folding and covalent modifications. The endoplasmic reticulum–associated protein degradation (ERAD) pathway is an evolutionarily conserved process that discards misfolded ER proteins (Wu and Rapoport, 2018). Three different ERAD pathways (ERAD-L, ERAD-M, and ERAD-C) are used for degrading misfolded ER proteins, depending on whether their misfolded domain is localized in the ER lumen, within the membrane, or on the cytosolic side of the ER membrane, respectively (Huyer et al., 2004; Vashist and Ng, 2004; Carvalho et al., 2006). A fourth pathway is responsible for misfolded protein removal from the inner nuclear membrane (Foresti et al., 2014; Khmelinskii et al., 2014). Each pathway involves distinct ubiquitin ligases and cofactors, although it is unclear how proteins are targets to each pathway. ERAD substrates from all pathways are retrotranslocated to the cytosolic side of the membrane (Wu and Rapoport, 2018). With the help of ubiquitination machinery and the Cdc48/p97 ATPase complex, the substrates are extracted from the membrane and delivered to the 26S proteasome for degradation (Bays et al., 2001; Braun et al., 2002; Jarosch et al., 2002; Rabinovich et al., 2002). Proteasome inhibition stalls ERAD and causes misfolded proteins to accumulate within the ER. In response, the cell activates a highly conserved signaling pathway called the unfolded protein response (UPR) (Tsai and Weissman, 2010). Multiple physiologic conditions also lead to accumulation of misfolded proteins in the ER and subsequent UPR activation, including hypoxia, glucose deprivation, oxidative stress, and mutations in certain secretory proteins (Tsai and Weissman, 2010). UPR activation regulates the gene expression involved in protein folding (e.g., chaperones) and ERAD and decreases protein translation into the ER in an attempt to restore ER homeostasis (Travers et al., 2000). The UPR initially performs a protective role in the cell. However, prolonged ER stress and UPR activation eventually leads to cell death (Travers et al., 2000). Wu and Rapoport (2018) recently published an extensive review discussing the molecular mechanisms of ERAD and associated protein degradation.

## III. Development of Proteasome Inhibitors

### A. The Rise of Proteasome Inhibitors

Our understanding of the importance of the UPS for biologic functions and processes rapidly advanced with the introduction of the first proteasome inhibitors (Rock et al., 1994). These valuable tools allowed researchers to interrogate proteasome function in complex cellular systems and tissues and greatly advanced our understanding of many aspects of cell regulation, disease mechanisms, and immune surveillance (Rock and Goldberg, 1999). Perhaps the most clinically important developments to come from the early proteasome inhibitor studies were advancements in understanding the regulation of NF-*κ*B and its key role in the pathogenesis of many inflammatory and neoplastic diseases (Palombella et al., 1994; Goldberg, 2007). The first proteasome inhibitors were simple hydrophobic peptide aldehydes (analogs of serine protease inhibitors) designed to mimic the preferred substrates of the proteasome’s chymotrypsin-like site (*β*5) and inhibit it (Goldberg, 2007). The tripeptide aldehyde, MG-132 (carbobenzyl-Leu-Leu-Leu-aldehyde), is still the most widely used proteasome inhibitor in scientific research because it is potent, inexpensive, and quickly reversible (Goldberg, 2007). Given the indispensable role of the proteasome in the NF-*κ*B pathway, proteasome inhibitors showed therapeutic potential for the treatment of some human diseases, yet it was also appreciated that complete proteasome inhibition would lead to cell death (Goldberg, 2007).

**Fig. 4.**
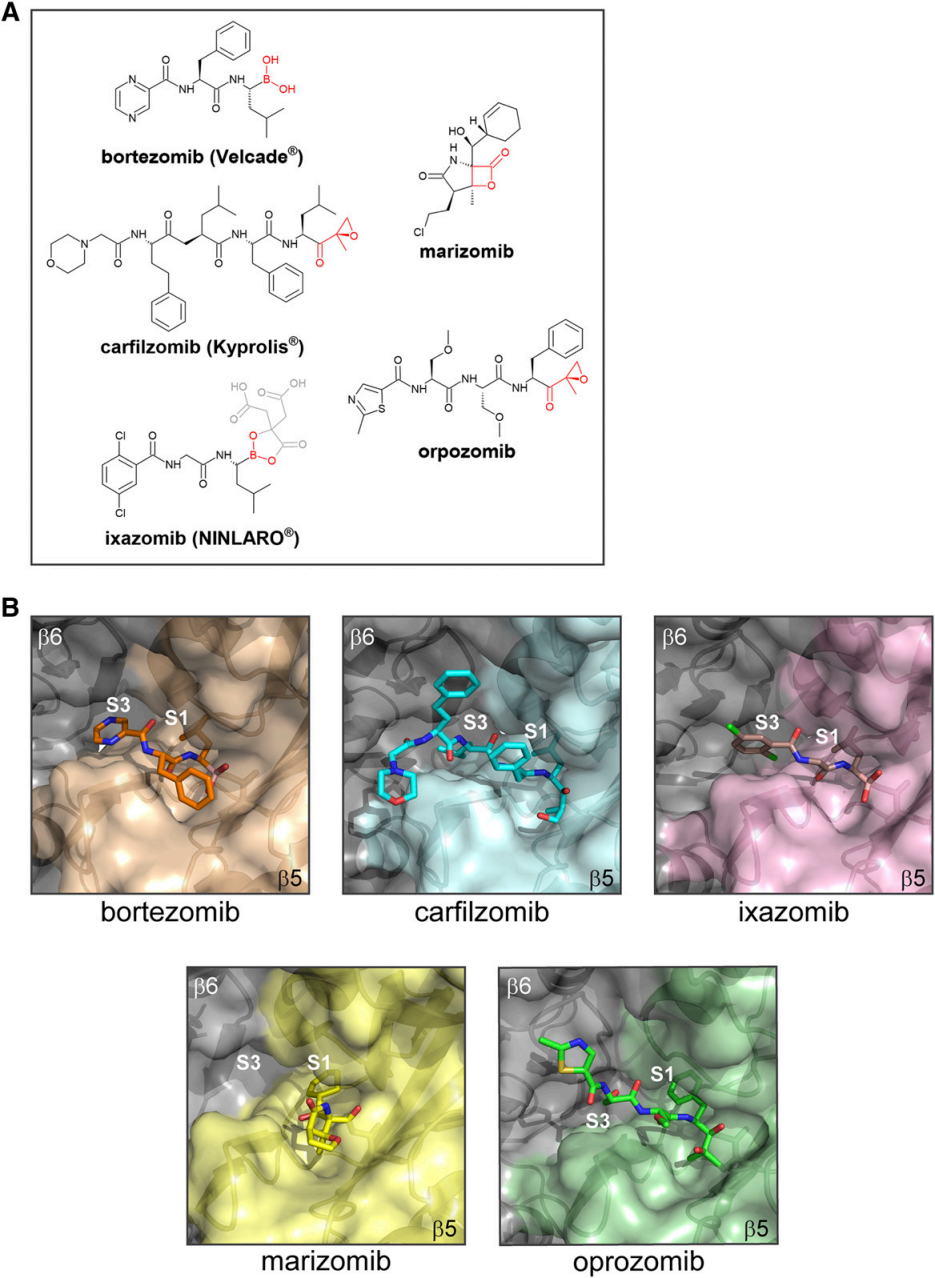
Proteasome inhibitors and the *β*5 binding site. (A) Chemical structures of proteasome inhibitors that are FDA approved and/or are in clinical trials with pharmacophores shown in red. For ixazomib, the orally bioavailable prodrug (MLN9708) is shown, with the biologically active metabolite (MLN2238) highlighted in black and red for clarity. (B) X-ray crystallography structures of human 20S proteasomes in complex with carfilzomib (PDB 4r67), bortezomib (PDB 5lf3), and ixazomib (PDB 5lf7); yeast 20S in complex with marizomib (PDB 2fak); and the cryo-EM structure of human 26S in complex with oprozomib (PDB 5m32). PDB, Protein Data Bank.

Researchers hypothesized that partial and reversible inhibition of the proteasome might be beneficial in killing neoplastic cells because they lack many of the checkpoint mechanisms that protect normal cells from apoptosis (Adams, 2001; Goldberg, 2007). Accordingly, proteasome inhibitors were preferentially toxic to transformed and patient-derived malignant cell cultures rather than their nontransformed and healthy counterparts (Masdehors et al., 1999; Adams, 2001). Aldehyde proteasome inhibitors (e.g., MG-132) had limited therapeutic potential in humans due to off-target effects (e.g., inhibition of cathepsin B and calpains) and poor metabolic stability (Adams et al., 1998b). With MG-132 as a lead compound, a team of medicinal chemists led by Julian Adams synthesized the dipeptide boronic acid PS-341 (Pyz-Phe-boroLeu), a slowly reversible inhibitor of the *β*5 active site (with some activity toward the *β*2 active site). PS-341 proved to be a potent and selective proteasome inhibitor, demonstrating therapeutic activity in preclinical models of inflammatory diseases and human cancers (An et al., 1998; Masdehors et al., 1999; Adams, 2001). PS-341 entered phase I clinical trials, in which remarkably one patient with MM showed a complete response to PS-341 treatment (Adams, 2001). MM is an incurable plasma cell malignancy; at that time, patients had a poor prognosis due to lack of effective treatment options, making the complete response to PS-341 a dramatic clinical breakthrough (Goldberg, 2007). PS-341 progressed to phase II trials for MM and chronic lymphoid leukemia. Due to the remarkable success of PS-341 in phase II trials, the FDA approved PS-341 (later renamed bortezomib and marketed as Velcade) (Fig. 4A) as a third-line treatment for relapsed and refractory multiple myeloma (RRMM) in 2003 (Goldberg, 2007). Bortezomib revolutionized the treatment of MM, and today bortezomib is approved for use as a first-line therapy for MM and mantle cell lymphoma.

Despite its clinical success in treating hematologic diseases, bortezomib therapy is associated with a high rate of resistance (primary or secondary) and serious dose-limiting toxicity, which require reduction or discontinuation of the drug (Goldberg, 2012). Advances in proteasome inhibitor chemistry and a better understanding of the proteasome’s unique catalytic mechanism have led to the development of second-generation proteasome inhibitors with improved pharmacokinetics compared with bortezomib (Goldberg, 2016). The mechanisms of available proteasome inhibitors and their uses in research and clinical settings are discussed in the following sections.

### B. Chemical Classes of Proteasome Inhibitors

There are several classes of proteasome inhibitors. Like the majority of protease inhibitors, most proteasome inhibitors are short peptides designed to fit into the substrate binding site on the catalytic subunit. The activity of proteasome inhibitors depends on the pharmacophore warhead at the C terminus, which reacts with the active site threonine nucleophile to form reversible or irreversible covalent adducts (Kisselev and Goldberg, 2001). Although the proteasome has three types of catalytic sites, inhibition of all three is not required to significantly affect protein degradation (Kisselev et al., 2006). Specific *β*1 or *β*2 inhibition does not have a significant effect on overall protein breakdown; however, *β*5 inhibition results in significantly reduced protein breakdown (Kisselev et al., 2006). Consequently, most proteasome inhibitors target the *β*5 site, although they often have some lesser activity against *β*1 and/or *β*2 (Kisselev et al., 2006).

#### 1. Peptide Aldehydes

Based on the well characterized serine and cysteine protease inhibitors, peptide aldehydes (e.g., MG-132) were the first synthesized proteasome inhibitors. MG-132 is cell permeable and is a reversible proteasome inhibitor, which makes it a valuable research tool. Because MG-132 has slow binding and fast dissociation kinetics (Kisselev and Goldberg, 2001), the effects of MG-132 proteasome inhibition on cultured cells are quickly reversed by switching to inhibitor-free media. The low cost and rapid reversibility make MG-132 the most used proteasome inhibitor for research (Goldberg, 2007). However, there are several limitations to MG-132. First, MG-132 in cell culture media is rapidly oxidized into an inactive acid (Kisselev and Goldberg, 2001). For long cell culture experiments, MG-132–containing media should be replaced daily. Second, as with other peptide aldehydes, MG-132 also inhibits (albeit with much lower affinity) calpains and cathepsins (Kisselev and Goldberg, 2001); therefore, it is necessary to perform control experiments to confirm that the observed effects are due to proteasome inhibition. Proteasome involvement can be verified using a more selective proteasome inhibitor (e.g., epoxomicin, boronates, and lactacystin), although these compounds may be cost prohibitive for routine studies or screens. In addition, inhibitors that specifically block other proteases, such as E-64 [(2*S*,3*S*)-3-[[(2*S*)-1-[4-(diaminomethylideneamino)butylamino]-4-methyl-1-oxopentan-2-yl]carbamoyl]oxirane-2-carboxylic acid] for calpains, but not the proteasome can be used to confirm that the observed effect is not due to off-target inhibition of another protease.

#### 2. Peptide Boronates

Peptide boronates are significantly more potent proteasome inhibitors compared with peptide aldehydes. Like peptide aldehydes, peptide boronates form a tetrahedral adduct with the active site threonine but their dissociation rate is much slower, making boronates practically irreversible over hour-scale time courses (Kisselev and Goldberg, 2001). MG-262 (Cbz-Leu-Leu-Leu-B(OH)2), the boronate analogue of MG-132, is 100-fold more potent than its aldehyde counterpart (Kisselev and Goldberg, 2001). In addition, peptide boronates are not oxidized into inactive forms like MG-132, making them more stable in vivo (Kisselev and Goldberg, 2001). The boronate warhead cannot react with active site cysteines, so they have fewer nonproteasome targets (Kisselev and Goldberg, 2001).

#### 3. Epoxomicin and Epoxyketones

Another naturally derived proteasome inhibitor is epoxomicin, an actinomycete fermentation metabolite. It is a modified peptide that contains a C-terminal *α*′,*β*′-epoxyketone group attached to an aliphatic P1 amino acid (Kisselev and Goldberg, 2001). Epoxomicin is extremely specific for the proteasome. The crystal structure of the yeast 20S proteasome in complex with epoxomicin revealed its unusual mechanism of action and explained the basis for proteasome specificity (Groll et al., 2000). Epoxomicin reacts covalently with both the catalytically active N-terminal threonine hydroxyl and the free amino group, producing a highly stable and irreversible morpholino ring (Groll et al., 2000). Unlike other proteasome inhibitors that only form a bond with the threonine hydroxyl, the double covalent bond formation of the epoxyketone group limits its reactivity to the N-terminal nucleophile threonine proteases without inhibiting any other cellular protease (Groll and Huber, 2004). Since the discovery of epoxomicin as a proteasome inhibitor, many *α*′,*β*′ epoxyketone electrophiles have been incorporated into peptide sequences and optimized for binding to the proteasome *β* subunits. The most well characterized epoxyketone inhibitor is carfilzomib (Kyprolis; Onyx Pharmaceuticals/Takeda, South San Francisco, CA) (Fig. 4A), a second-generation, FDA-approved proteasome inhibitor for treatment of RRMM (discussed further in section V).

#### 4. Lactacystin and β-Lactone

Lactacystin, a *Streptomyces* metabolite, is a nonpeptide proteasome inhibitor. Lactacystin itself does not inhibit the proteasome, but at neutral pH lactacystin spontaneously converts to clasto-lactacystin-*β*-lactone, which is reactive with the proteasome. The *β*-lactone reacts with the proteasome active site threonine, resulting in opening of the *β*-lactone ring and acylation of the proteasome catalytic threonine hydroxyl (Groll and Huber, 2004). The yeast 20S proteasome in complex with the lactacystin crystal structure confirmed this mechanism, providing strong evidence that an acyl enzyme conjugate is an intermediate in proteasome catalysis (Groll and Huber, 2004). Lactacystin is more proteasome specific than MG-132, with a single off-target substrate (cathepsin A). Although lactacystin is considered an irreversible proteasome inhibitor, its adduct is slowly water hydrolyzed (half-life of approximately 20 hours). Lactacystin is the least stable of the proteasome inhibitors and exists in vivo in equilibrium with lactathione, its glutathione reaction product (Kisselev and Goldberg, 2001). Despite this drawback, the high proteasome selectivity makes lactacystin a viable proteasome inhibitor for investigating the role of the proteasome in cellular processes.

#### 5. Vinyl Sulfones

Peptide vinyl sulfones are a class of irreversible proteasome inhibitors. Peptide vinyl sulfones also inhibit cysteine proteases (e.g., cathepsins), but changing the functional groups in the inhibitor’s peptide portion can modulate their specificity (Screen et al., 2010). For example, replacing the benzyloxycarbonyl (*Z*) group with the 3-nitro-4-hydroxy-5-iodophenylacetate group in ZLVS (*Z*-Leu3-VS) generates NLVS, significantly reducing inhibition of cathepsins B and S (Kisselev and Goldberg, 2001). Peptide vinyl sulfones are easy and inexpensive to synthesize, and their irreversible binding makes them attractive as protease activity probes. Peptide vinyl sulfones are commonly labeled with fluorophores, biotin, or a radioactive moiety, and specific uses as proteasome activity probes are discussed in section IV. Interestingly, vinyl sulfones are more potent and more trypsin-like site–selective inhibitors than epoxyketones with an identical peptide sequence (Kraus et al., 2015), a feature exploited in the development of *β*2-specific proteasome inhibitors, as discussed in section V.

### C. Considerations for Proteasome Inhibitor Design

In this section, we review the structural features that have been exploited to design specific inhibitors of the 20S catalytic sites.

#### 1. Substrate Binding Pockets

All six proteasome catalytic subunits (constitutive and immune) have a similar substrate binding site topology, in which the S1 position is buried in the subunit next to the threonine, the S2 is solvent exposed, and both the catalytic subunit and its neighbor contribute to the S3 binding position (Groll and Huber, 2003) (Fig. 4B). However, residues that make up the S1 and S3 sites have very different catalytic subunit properties. Modification of the P1 and P3 sites on a proteasome inhibitor can significantly alter their subunit specificity and affinity. *β*5c prefers a small hydrophobic group in P1 and a large hydrophobic group in P3, whereas *β*5i favors the inverse arrangement (Groll and Huber, 2004). Therefore, altering the hydrophobic group size in P1 and P3 confers selectivity for *β*5c or *β*5i. Due to solvent exposure in the P2 position, P2 can accommodate a range of moieties without affecting proteasome binding and is often the site for modifications aimed at improving inhibitor solubility and stability. P2 is also a useful attachment site for fluorescent probes, biotin tags, or azide handles (discussed further below).

#### 2. Structural Analysis of Bound Inhibitors

Knowledge of protein structure and its interaction with ligands guides drug discovery and design. X-ray crystallography is an excellent method for obtaining high-resolution proteasome structures in complex with inhibitors and has been instrumental in understanding proteasome function and advancing proteasome inhibitor development (Borissenko and Groll, 2007). The early structures of ALLN- and lactacystin-bound proteasomes provided clues as to the threonine catalytic mechanism and intermediate states (Borissenko and Groll, 2007). The structures of a substrate analog-bound proteasome showed long-range allosteric changes that occur upon substrate binding in the active site. The inhibitor-bound structure can be used together with biochemical data for structure-activity relationship studies and subsequent lead compound optimization.

There are drawbacks to using crystallography to study proteasome inhibitor–proteasome interactions. First, solvent conditions and inhibitor concentrations used in cocrystallization are not physiologic and should be considered when interpreting the resultant structure. 20S proteasome crystals are usually soaked in solutions with high inhibitor concentrations under conditions that preserve crystal integrity, whereas enzymatic assays are carried out at 37°C with low inhibitor concentrations and proteasomes under conditions optimized for substrate degradation. Discordant data in the yeast 20S proteasome structure in complex with ALLN showed that the inhibitor bound to all six proteasome active sites, but the biochemical data indicated that the ALLN proteasome inhibitor preferentially inhibited the *β*5 site (with very low activity against *β*1 and *β*2) unless used at extremely high concentrations (Groll and Huber, 2004). If one considers that most proteasome inhibitors affect multiple active sites at high concentrations and that proteasome inhibitor concentration in the crystallization condition was in the millimolar range, it is unsurprising that the inhibitor bound all sites. This example illustrates the need to carefully consider existing biochemical data when interpreting new structures.

In addition, obtaining good diffraction data relies on homogenous crystal packing. Under these conditions, one may miss larger-scale conformational changes that take place upon 20S proteasome ligand binding. The inherent drawbacks in crystallography methodology highlight the need to incorporate other structural methods into understanding the proteasome. Recent advances in cryo-electron microscopy (EM) and single particle analysis make it possible to obtain near atomic level–resolution protein structures in more physiologic conditions (da Fonseca and Morris, 2015). Proof of principle for the cryo-EM utility in structure-based drug discovery and development is found in Morris and da Fonseca (2017). Recently, the noncovalent reversible asparagine-ethylenediamine (AsnEDA)-based, inhibitor-bound human immunoproteasome cryo-EM structure was used in structure-activity relationship studies (Santos et al., 2017). Exploiting *β*5c/*β*5i residue differences near the S1 pocket improved PKS21187 (AsnEDA-based inhibitor) affinity for *β*5i down to 15 nM (from 58 nM) and successfully improved selectivity (20-fold) over *β*5c (Santos et al., 2017). Although cryo-EM typically cannot provide quite the same resolution as crystallography, the 20S core particle characteristically has high local resolution (approximately 3 Å, in both of the above studies) at the interior of the *β* subunits, making this a valuable method for investigating proteasome ligand binding under relatively physiologic conditions.

It is important to keep in mind that cryo-EM structures are derived from averaging classes of particles, meaning that protein subconformations may be overlooked. This is important in interpreting 26S proteasome structures because the complex undergoes large-scale conformational changes during cycles of substrate binding and ATP hydrolysis, resulting in many conformational states coexisting simultaneously. Despite physiologically relevant conditions, conformer subpopulations may not be apparent. For example, Baumeister and colleagues published 26S proteasome in the ATP-hydrolyzing state (Beck et al., 2012) and the adenosine 5′-[*γ*-thio]-triphosphate–bound cryo-EM structures (Śledź et al., 2013). However, when they performed a deep classification of more than 3 million 26S proteasome particles in the presence of both ATP and adenosine 5′-[*γ*-thio]-triphosphate, they identified a third state of the 26S proteasome that was believed to be an intermediate conformation during the ATP hydrolysis cycle (Unverdorben et al., 2014). Additional intermediate 26S conformation states have been identified in humans and yeast using cryo-EM (Chen et al., 2016; Wehmer et al., 2017; Guo et al., 2018).

#### 3. Proteasome Inhibitor Pharmacophore Properties

As previously discussed, pharmacophores confer specific proteasome inhibitor properties, including compound stability, off-target protease inhibition, and inhibition kinetics. Interestingly, the pharmacophore nature is suggested to influence proteasome inhibitor active site specificity. Epoxyketone warhead replacement with vinyl sulfone moieties in *β*5 inhibitors further improves *β*5 site (but not *β*5i site) selectivity (Screen et al., 2010). Therefore, each warhead confers unique properties to the proteasome inhibitor; thus, selecting a pharmacophore along with the appropriate controls requires careful consideration.

## IV. Methods for Pharmacological Proteasome Research

Extensive methodology exists for investigating proteasome function in vitro and in vivo. Here, we describe some commonly used methods for proteasome purification, peptidase activity assays, and protein degradation assays in vitro and in cell culture, and we discuss their advantages and limitations.

### A. Proteasome Purifications

Rigorous and reproducible studies of proteasome pharmacology require a source of pure and active proteasomes. The following is a summary of methods for endogenous and affinity-tagged proteasome purifications.

#### 1. Endogenous Proteasome Purification

Endogenous proteasomes are purified from a variety of tissues using a series of anion exchange chromatography columns (Kisselev et al., 2002; Smith et al., 2005). After purification, 20S and 26S proteasomes are separated by gel filtration or glycerol gradient centrifugation. Because 26S proteasomes require ATP binding to remain intact, omitting ATP from the homogenization and chromatography buffers enriches for 20S proteasomes. Rabbit skeletal muscle and bovine liver have abundant proteasomes, making it possible to obtain mostly pure proteasomes (>95%) in milligram quantities in under a week with anion exchange chromatography.

Another method to purify endogenous 26S proteasomes from almost any tissue or cell type takes advantage of the 19S regulatory particle’s affinity for proteins containing UBL domains (Besche and Goldberg, 2012). Recombinant glutathione *S*-transferase (GST) fused to the RAD23B UBL domain, a ubiquitin shuttling factor, is purified from *Escherichia coli* and bound to glutathione beads. 26S proteasomes in cell or tissue lysates bind the GST-UBL column while all other cellular proteins are washed away. Bound 26S proteasomes are subsequently eluted with high concentrations of a tandem ubiquitin-interacting motif derived from Rpn10, a 19S ubiquitin binding subunit (Besche and Goldberg, 2012). Unlike anion exchange chromatography (which takes approximately 3 days), the UBL-affinity method takes a single day and does not use high-salt buffers. This rapid and gentle 26S purification is essential to retain loosely associated proteasome proteins that are lost during anion exchange chromatography.

Because the GST-UBL bait occupies UBL binding sites on 19S, endogenous UBL domain-containing proteins and ubiquitin conjugates may be dislodged from the proteasome upon purification (Kuo et al., 2018). Despite this limitation, the UBL-affinity method has been valuable in studies investigating 26S proteasome composition in a variety of physiologic and disease states. For example, Qiu et al. (2006) used the UBL-affinity method to identify Rpn13, a novel human 19S subunit that tethers and activates UCHL5 (a DUB) to the 26S proteasome and functions as a ubiquitin receptor (Husnjak et al., 2008). The UBL-affinity method also copurifies other important proteasome-associated proteins with important roles in regulating proteasome function and ubiquitin-conjugate degradation (e.g., the DUB USP14) (Kuo et al., 2018). A significant advantage of the UBL-affinity method is that one can purify proteasomes from diseased tissues and study the changes in proteasome activity and composition without genetic alterations.

Most commercially available 20S and 26S proteasomes are purified from mammalian (e.g., human, rabbit) erythrocytes. Since erythrocytes lack nuclei, these endogenous proteasomes are “aged,” perhaps with oxidative damage, and may have lower basal activity and poorer gating function than those derived from nucleated cells.

#### 2. Affinity-Tagged Proteasomes

Several groups have created human cell lines and yeast strains stably expressing affinity-tagged proteasome subunits to rapidly isolate proteasomes for structural and functional studies. Affinity tags are often appended to the Rpn11 or *β*4 C termini because modifications on these subunits do not effect proteasome function in cellular or yeast cultures.

Affinity-tagged proteasome purifications are especially useful for studying changes in 26S proteasome composition because they typically copurify with more ubiquitin conjugates and proteasome-interacting proteins than other methods. For example, Leggett et al. (2002) used a tobacco etch virus (TEV) protease–cleavable, protein A–derived tag on Rpn11, Rpt1, and *β*4 to study proteasome-interacting protein regulation of yeast 26S complex stability. The high purity and yield of affinity-tagged proteasomes is well suited for cryo-EM studies. For example, Matyskiela et al. (2013) used cryo-EM analysis of Rpn11-3xFLAG yeast proteasomes to study the conformational dynamics during 26S substrate engagement. Affinity tags are also amenable to high purification efficiency of crosslinked complexes under fully denaturing conditions. Guerrero et al. (2006) designed a tandem affinity tag consisting of a hexahistidine sequence followed by an in vivo biotinylation signal, termed HB. Tandem affinity purification of Rpn11-HB proteasomes after in vivo crosslinking combined with tandem mass spectrometry and quantitative stable isotope labeling of amino acids in cell culture enabled global mapping of the 26S proteasome interaction network in yeast (Guerrero et al., 2006). A TEV-cleavable version of the HB tag (HTBH and HBTH) allows for one-step purification of human 26S proteasomes. Wang et al. (2017) generated several stable human embryonic kidney 293 (HEK293) cell lines expressing tagged subunits (e.g., Rpn11-HTBH, HBTH-Rpn1, HBTH-Rpt6). Utilizing these cell lines with in vivo and in vitro crosslinking mass spectrometry workflows and cryo-EM approaches allows comprehensive examination of protein–protein interactions within the 26S proteasome (Wang et al., 2017). Choi et al. (2016) developed stable *β*4-HTBH/*α*3-FLAG and *β*4-HTBH/*α*3ΔN-FLAG HEK293 cell lines. The *α*3 N-terminal deletion (*α*3ΔN) results in a constitutively open gate. These cell lines are a tool for investigating the role of dynamic proteasome gating and are discussed further in section VI. It is worth noting that these cell lines stably express the tagged subunit in addition to the endogenous gene; thus, purification does not isolate all proteasome complexes and may not reflect all changes in proteasome composition under different physiologic states or drug treatments.

#### 3. Immunoproteasomes

Immunoproteasome preparations are usually purified from spleens and cell cultures, treated with IFN-*γ*, to increase immunoproteasome subunit expression. Immunoproteasomes can be purified using the same anion exchange chromatography methods as described above for constitutive proteasomes. However, constitutive proteasomes are ubiquitously present in all tissue types and even low amounts can interfere with immunoproteasome-specific research. Dechavanne et al. (2013) report that hydrophobic interaction chromatography can successfully separate immunoproteasomes from residual constitutive proteasome contamination after purification.

#### 4. Validating and Storing Proteasome Preparations

It is imperative to check for contaminating proteases in all proteasome preparations. For example, proteasomes can copurify with tripeptidyl-protease II (TPP-II) during anion exchange chromatography. TPP-II is a serine protease capable of cleaving the peptide substrates used in proteasome activity assays; thus, TPP-II peptidase activity should not be confused with that of the proteasome. Proteasome-specific activity can be confirmed with proteasome inhibitors as a negative control.

Regardless of the purification method, it is necessary to confirm the 20S/26S proteasome assemblies after preparation. For example, purifying affinity-tagged RPN11 proteasomes will select for single-capped 20S, double-capped 20S, and free 19S particles, whereas affinity-tagged 20S subunits select for free 20S, 26S, and 20S associated with other regulators (e.g., PA28, PA200). 20S proteasomes and 20S bound to regulators are clearly separated by native PAGE (e.g., NuPAGE 3%–8% Tris-Acetate Gel; Thermo Fisher Scientific, Waltham, MA) (Besche and Goldberg, 2012). Electrophoresis with 26S proteasomes is performed with adequate ATP and MgCl_2_ in the buffer to prevent complex dissociation and kept at 4°C (Table 2). After electrophoresis, peptidase activity of proteasome complexes is measured by an in-gel fluorescence activity assay (Besche and Goldberg, 2012). The gel is incubated at 37°C in buffer containing the fluorogenic peptide substrate succinyl-leucine-leucine-valine-tyrosine (Suc-LLVY)-7–amino‐4‐methylcoumarin (amc) and the cleaved amc fluorophore is visualized with UV light. The addition of 0.02% SDS to the gel incubation buffer enhances 20S peptidase activity and improves visualization of the 20S band. After UV imaging, the gel can be processed with Coomassie or silver stain, analyzed by two-dimensional native SDS-PAGE, or transferred to a membrane for immunoblot analysis. Due to the large size of the proteasome complexes (700 kDa to 2.4 MDa), incubating the gel in SDS buffer prior to transfer may improve transfer efficiency and increase epitope availability. Roelofs et al. (2018) provide methods for various downstream analyses to investigate the activity and composition of proteasome complexes separated by native PAGE.

The association between 19S and 20S is labile and sensitive to changes in temperature and nucleotide presence. To maintain 26S proteasome complex integrity, purification should be performed as rapidly as possible in the presence of adequate ATP and MgCl_2_ and kept at 4°C to prevent the hydrolysis of ATP. The addition of glycerol (approximately 10%) in purification and storage buffers stabilizes 26S complexes and 20S gate latency (i.e., gating function). After purification, 20S and 26S proteasomes are typically flash frozen in liquid nitrogen and stored at −80°C. Further freeze/thaw cycles should be avoided, as this damages the proteasome and affects its activity. It is important to thaw frozen 26S proteasomes on ice and use them immediately after thawing. Since freezing, storage conditions, and thawing can affect the labile 26S proteasome assembly, it is recommended that one verify 26S proteasome complexes after thawing with native PAGE.

### B. Monitoring Proteasome Activity

There are numerous methods for monitoring proteasome activity in vitro and in vivo. In vitro experiments are performed with either peptide- or protein-based model substrates. The following section covers commonly used peptide- and protein-based model proteasome substrates and methods for monitoring their degradation. Finally, we discuss artificial proteasome substrates for expression in cell culture and transgenic animals.

#### 1. Peptide-Based Model Substrates

In vitro proteasome activity is often measured using a fluorescent substrate enzyme activity assay. Peptide substrates are useful for monitoring proteasome gating and peptidase activities and are amenable to high-throughput formats. Model peptide substrates are short tri- or tetrapeptides with a C-terminal fluorophore (e.g., amc).

Amino acid sequences are designed to preferentially interact with and be degraded by specific 20S subunits (Fig. 2B; Table 1). Common peptide substrates are Suc-LLVY-amc, *tert*-butoxycarbonyl-leucine-arginine-arginine-amc, and *N*-acetyl-norleucinal-proline-norleucinal-aspartate-amc for *β*5 chymotrypsin-like, *β*2 trypsin-like, and *β*1 caspase-like activities, respectively.

**TABLE 1:** Fluorogenic peptides

Subunit	Preference	Substrate
*β*1	Acidic	Ac-nLPnLD-amc
		*Z*-LLE-amc
*β1i*	Hydrophobic	Ac-PAL-amc*^a^*
*β*2	Basic	Boc-LRR-amc
		Ac-RLR-amc
*β*2i	Basic	Not applicable*^b^*
*β*5	Hydrophobic	Suc-LLVY-amc
		Ac-WLA-amc
*β5i*	Hydrophobic	Ac-ANW-amc*^a^*

Ac-nLPnLD-aminoluciferin, *N*-acetyl-norleucinal-proline-norleucinal-aspartate-amc; Boc-LRR, *tert*-butoxycarbonyl-leucine-arginine-arginine.

^a^ These substrates differentiate between *β*5 and *β*5i activity.

^b^ Substrates specific for *β*2i have not been developed to date.

When the amc moiety is covalently attached to the peptide, its fluorescence (excitation, 380 nm; emission, 460 nm) is greatly decreased compared with free amc. Proteolytic cleavage releases amc from the peptide and the resulting increase in fluorescence intensity is directly proportional to proteasome proteolytic activity. Fluorescence intensity is monitored in real time with a microplate reader and the rate of cleavage is determined from the slope of the reaction progress curve. This assay is rapid and suitable for high-throughput studies. Cell-based reagent kits use similar aminoluciferin-fused peptide substrates, which allows proteasome peptidase activity measurement in intact cells.

Although fluorescent substrate peptides are an excellent tool for preliminary studies measuring proteasome activity (e.g., screening compound libraries), there are several pitfalls (Table 2). Foremost, small peptides bypass the need for 19S recognition and unfolding and thus only report 20S peptidase activities. Therefore, changes in peptide degradation do not necessarily translate into changes in protein degradation. Importantly, at high peptide substrate concentrations, multiple active site types can participate in peptide cleavage. As such, chymotrypsin-like (*β*5) activity (Suc-LLVY-amc) assays evaluating response to a proteasome inhibitor may overestimate the reduction in protein degradation in vivo. Therefore, multiple substrate types may be required to evaluate experimental changes in proteasome activity, as various proteasomal forms (compositions) will elicit different activities to the different types of substrates.

**TABLE 2:** Experimental pitfalls

Experimental variables	Pitfall
Buffer	
ATP and magnesium	Stability of the 26S complex is dependent on a high ATP/ADP ratio. For monitoring 26S activity, an adequate amount of ATP and MgCl_2_ in a 1:5 ratio (we typically use 2 and 10 mM, respectively) should be present in all buffers to maintain the stability of the 26S proteasome complex. At the same time, the levels of ADP should be kept at a minimum
Glycerol	Glycerol in the buffer stabilizes and maintains the closed, latent gate. Typically, purification buffers for proteasomes contain 10% glycerol, and assay buffers contain 5%
SDS	Addition of 0.02% SDS to assay buffer mildly stimulates opening of the latent 20S gate
Proteasome population	Proteasomes are present as individual 20S particles and 26S particles (singly caped 20S-19S and doubly capped 19S-20S-19S). The 26S complex can dissociate into the 19S and 20S constituents over time, after a freeze/thaw, or in response to buffer condition changes
	When performing experiments with purified 26S proteasomes, it is important to determine the ratio of intact 26S and free 20S proteasomes. This is commonly accomplished by native PAGE electrophoresis as described in the text
Microplate	The type of microplate (e.g., untreated vs. nonbinding surface treatments, polypropylene vs. polystyrene) can affect the observed activity. Some substrates (e.g., GFP) interact with and “stick” to untreated plate surfaces. Some compounds or small substrates may bind to the plate surface and reduce the effective concentration in the assay. It is worthwhile to confirm results with new compounds or substrates by using two or more types of plates. Bovine serum albumin can be included in the buffer to prevent nonspecific binding to the plate
Proteasome gating	Experiments probing the role/regulation of the proteasome gate might be facilitated by using the “open-gate” 20S mutant, *α*3ΔN
	Suc-LLVY-amc has been shown to mildly stimulate gate opening, so another substrate (e.g., Ac-nLPnLD-amc) should be used in conjunction

#### 2. Protein-Based Model Substrates

Many protein substrates are available to monitor 20S and 26S proteasome degradation in vitro (e.g., using purified proteasomes or cellular lysates). Here we describe methods for quantitating protein degradation and give examples of model substrates for 20S and 26S proteasome activity.

##### a. Methods to quantitate protein degradation

The extent of in vitro protein degradation can be measured in several ways. The fluorescamine assay is a quantitative method for measuring protein cleavage products generated by the proteasome. The proteasome processively degrades proteins and the generated products are equivalent to the number of substrate molecules degraded multiplied by the mean number of cuts made in a single polypeptide (Kisselev et al., 2006). Fluorescamine addition to an amine free assay buffer quickly labels new N-terminal amines on proteolytically cleaved short peptides.

Alternatively, the proteins can be separated by SDS-PAGE and monitored for substrate band disappearance via Coomassie or silver staining or immunoblot analysis. Care must be taken when monitoring degradation via Western blot analysis, as degradation of the epitope results in complete loss of signal, which may not correlate with complete protein degradation. Since most gel-based protein degradation assays are performed with small reaction volumes (<20 *µ*l) incubated in centrifuge tubes, the amount of substrate remaining should be normalized to a loading control (e.g., a 20S proteasome subunit) to control for loss of substrate protein during pipetting or incubation steps. Fluorescence anisotropy is useful to follow 20S and 26S proteasome degradation of fluorescent dye–labeled protein substrates in real time (Bhattacharyya et al., 2016; Thibaudeau et al., 2018). Singh Gautam et al. (2018) describe methods for high-throughput measurement of 26S ubiquitin-dependent degradation using dye-labeled substrates.

##### b. 20S (19S-independent) substrates

Intrinsically disordered proteins are unstructured, thus abrogating the requirement for the unfoldase activity associated with the 19S regulatory particle. *β*-casein is a good substrate for monitoring 20S protein degradation and is commercially available. One should use aggregation-prone proteins (e.g., *α*-synuclein) with caution, since soluble oligomers can impair proteasome function (Thibaudeau et al., 2018).

##### c. 26S (ubiquitin-independent) substrates

Folded substrates are required to determine the 19S contribution toward 26S proteasome degradation. Ornithine decarboxylase (ODC) is a stably folded protein containing a C-terminal degradation tag (Ghoda et al., 1989) that promotes rapid ubiquitin-independent degradation by 26S proteasomes (Murakami et al., 1992). Fusing the ODC degradation tag (cODC) to other proteins promotes their proteasomal degradation (Hoyt et al., 2003). For example, cODC fusion to the titin I27 domain allows for ubiquitin-independent degradation of a folded protein, although the kinetics may be slow (Henderson et al., 2011). Destabilizing mutations can be introduced to I27 and accelerate substrate degradation (Henderson et al., 2011).

##### d. 26S (ubiquitin-dependent) substrates

The 26S ubiquitin-dependent degradation of folded proteins can be monitored with a tetra-ubiquitin fused green fluorescent protein (GFP) (Martinez-Fonts and Matouschek, 2016; Singh Gautam et al., 2018) with a C-terminal unstructured region (Prakash et al., 2004) or a tetra-ubiquitinated dihydrofolate reductase (Thrower et al., 2000). It is also possible to express some cODC fusion proteins in vivo to monitor proteasome activity.

#### *3. Proteasome Activity in Cell Culture*

The 26S proteasome degrades ubiquitinated proteins and proteasome impairment leads to polyubiquitinated protein accumulation. Measuring changes in high molecular weight polyubiquitin protein conjugates via immunodetection is used to monitor changes in proteasome activity. This is a general, nonspecific method to measure changes in proteasome degradation and should not be used in isolation to assess proteasome activity.

Stable GFP-fusion reporter expression is commonly used in cell culture to monitor proteasome activity. Measuring fluorescent reporters is a well-established technique for monitoring proteasome activity. Changes in reporter protein levels (in the absence of translation changes) inversely reflect UPS degradative capacity. Wild-type GFP has a long half-life in mammalian cells and therefore is not a suitable proteasome degradation substrate for most experiments. Several GFP fusion proteins have been engineered as specific proteasome substrates (Table 3). Bence et al. (2001) designed a synthetic reporter consisting of a short degron CL1, a consensus ubiquitination signal sequence first identified in fission yeast (Gilon et al., 1998), fused to the C terminus of GFP (GFP^u^), thereby targeting it for ubiquitin-dependent proteasome degradation. CL1 degron addition converted the GFP half-life from approximately 10 hours to 30 minutes. Cell compartment–specific proteasome function can be monitored by localization of GFP^u^ directed to the nucleus (nuclear localization signal-GFP^u^), the cytoplasm (nuclear export signal-GFP^u^) (Bence et al., 2005; Bennett et al., 2005), or neuronal synapses (postsynaptic density 95-GFP^u^ and synaptosomal-associated protein 25-GFP^u^) (Wang et al., 2008) (Table 3).

**TABLE 3:** Cellular proteasome substrates

Substrate	Ub/Pathway	Localization	*t*_1/2_	Reference
GFP^u^	Yes/CL1 degron		30 min	Bence et al. (2001)
NLS-GFP^u^	Yes/CL1 degron	Nuclear	60 min	Bennett et al. (2005)
NES-GFP^u^	Yes/CL1 degron	Cytosolic	60 min	Bennett et al. (2005)
PSD95-GFP^u^	Yes/CL1 degron	Postsynaptic	ND	Wang et al. (2008)
SNAP25-GFP^u^	Yes/CL1 degron	Presynaptic	ND	Wang et al. (2008)
TCR-*α*–GFP	Yes/ERAD		ND	DeLaBarre et al. (2006)
Ub-M-GFP	Yes/“normal”		“Stable”	Dantuma et al. (2000)
Ub-R-GFP	Yes/N-end rule		“Short”	Dantuma et al. (2000)
Ub^G76V^-GFP	Yes/UFD		“Short”	Dantuma et al. (2000)
Ub^G76V^-dendra2	Yes/UFD		ND	Hamer et al. (2010)
GFP-ODC	No		2 h	Li et al. (1998)

M, methionine; ND, not determined; NES, nuclear export signal; NLS, nuclear localization signal; PSD95, postsynaptic density 95; R, arginine; SNAP25, synaptosomal-associated protein 25; *t*_1/2_, in vivo half-life; TCR-*α*, T-cell receptor *α* chain.

Other UPS reporters have been generated to determine targeted proteasome degradation using different pathways. The N-end rule relates the cellular protein half-life to the identity of its N-terminal residue (Varshavsky et al., 1987). Fluorescent substrates of the N-end rule degradation pathway have been created with ubiquitin-GFP fusion constructs. When these ubiquitin fusion proteins are expressed in cells, DUBs rapidly cleave the ubiquitin and expose an unmodified N-terminal GFP residue. N-terminal arginine residue exposure (e.g., the substrate ubiquitin-R-GFP) recruits ubiquitin recognin box UBR domain E3 ligases that ubiquitinate the protein, targeting it for proteasome degradation (Dantuma et al., 2000). Techniques for generating ubiquitin fusion proteins with varying half-lives and conditional mutants are described in Dohmen and Varshavsky (2005) and Varshavsky (2005). Unlike ubiquitin-R-GFP, the reporter Ub^G76V^-GFP cannot be deubiquitinated (thereby bypassing the N-end rule pathway) and is a model substrate for the in vivo ubiquitin fusion degradation (UFD) pathway (Dantuma et al., 2000). The T-cell receptor protein *α* chain is rapidly degraded in nonhematopoietic cells, and a T-cell receptor protein *α*–GFP fusion protein can monitor ERAD-specific proteasome activity (DeLaBarre et al., 2006). Protein synthesis also influences the steady-state protein levels, and synthesis rates can be affected by cellular stress and transfection efficiency and vary from cell to cell. Therefore, it is imperative to use appropriate experimental design to take expression and translation differences into account when monitoring protein degradation. Commonly used methods are pulse-chase experiments (Sha et al., 2018), cycloheximide chase experiments (Chou and Deshaies, 2011), bicistronic expression vectors (Cadima-Couto et al., 2009), and measurement of stable long-lived proteins (Kisselev et al., 2006). Finally, each substrate only illustrates a single degradation pathway, which may or may not accurately reflect all UPS perturbations within a cell. It is advantageous to consider the use of multiple proteasome substrates to confirm findings.

In addition to cellular proteasomal activity, proteasome localization and dynamics can also be monitored with fluorescently labeled proteasome subunits. Both *α* and *β* subunits can be fused to a fluorescent protein and have been shown to efficiently incorporate into proteasome particles. For example, *α*4-yellow fluorescent protein (Otero et al., 2014) and a cyan fluorescent protein–tagged *β*1i (Reits et al., 2003) have been used to monitor localization dynamics of constitutive and immunoproteasomes in living cells. Detailed methods for monitoring proteasome dynamics in living cells are described elsewhere (Groothuis and Reits, 2005).

#### *4. In Vivo Proteasome Activity*

Transgenic animals that carry UFD proteasome substrates have also been generated and are used to study proteasome function in live tissues (Lindsten et al., 2003; Luker et al., 2003). Detailed methods for monitoring UFD protein degradation in yeast, cell lines, and transgenic mice are described in Menéndez-Benito et al. (2005). The photoactivatable Ub^G76V^-dendra2 construct monitors proteasome activity independent of translation and has been successfully used in transgenic *Caenorhabditis elegans* to determine tissue-specific proteasome degradation rates (Hamer et al., 2010).

### C. Proteasome Active Site Probes

Activity-based probes (ABPs) recognize catalytic sites on the constitutive or immunoproteasomes without requiring genetic techniques. Most proteasome ABPs are modified proteasome inhibitors with a fluorescent molecule incorporated at or near the N terminus. ABPs have been developed that can distinguish specific constitutive and immunoproteasome subunits (Hewings et al., 2017). After proteasome labeling and SDS-PAGE protein separation, the modified proteasome subunits are immediately visualized via in-gel fluorescence or immunoblotting. Furthermore, cell-permeable ABPs are compatible with live-cell imaging to detect real-time proteasome localization or with flow cytometry–based experiments. Site-selective ABPs are useful in determining novel proteasome inhibitor subunit specificity. A recent review (Hewings et al., 2017) provides a detailed account of currently available APBs.

## V. Proteasome Inhibitors to Treat Human Disease

### A. Hematologic Malignancies

#### 1. Bortezomib and Multiple Myeloma

At therapeutic doses, bortezomib inhibits approximately 30% of proteasome-mediated protein degradation (Adams, 2001), which is sufficient to induce MM tumor cell apoptosis without causing general toxicity in nontransformed cells. Considering the indispensable role of proteasome function in all cell types, this raises an important question: why is bortezomib particularly toxic to MM cells? First, proteasome inhibition stabilizes the NF-*κ*B complex in the cytoplasm and reduces NF-*κ*B–dependent gene expression. MM cells have increased NF-*κ*B activity and rely on this pathway for survival and proliferation (Hideshima et al., 2002). Furthermore, NF-*κ*B activity generates more proinflammatory NF-*κ*B activators in a positive feedback loop; therefore, partial proteasome inhibition is sufficient to reduce this pathologic cascade (Adams, 2003). Bortezomib-mediated NF-*κ*B inhibition enhances the effects of anti-MM conventional chemotherapeutic agents (e.g., dexamethasone, lenalidomide) and increases progression-free survival and overall survival for patients with MM (Goldberg, 2016). Second, proteasome inhibition reduces misfolded protein clearance. MM arises from mature Ig-secreting plasma cell hyperproliferation in the bone marrow and MM cells have a high rate of Ig production. Igs are large multisubunit molecules synthesized and folded in the ER, where they are post-translationally modified prior to secretion. The high Ig production rate and multiple modifications make MM cells heavily reliant on the proteasome and ERAD to maintain ER homeostasis (Chhabra, 2017); and they are more sensitive to proteasome inhibition. Accordingly, MM treatment with proteasome inhibitors results in a toxic misfolded protein buildup activating c-Jun N-terminal kinase and eventually resulting in apoptosis. Third, proteasome inhibition stabilizes various tumor suppressor proteins (e.g., p27, p53) and prevents cell cycle progression (Chen et al., 2007).

#### 2. Bortezomib Resistance

Although bortezomib revolutionized the treatment of MM, bortezomib resistance and relapse often occurs in patients who initially respond to bortezomib. Therefore, bortezomib resistance is a major issue for MM therapy. There are several mechanisms linked to bortezomib resistance and they fall into two broad categories. First, there are changes in proteasome subunit composition and expression (Lü et al., 2008). In addition, mutations in the *β*5 bortezomib binding pocket are associated with bortezomib resistance in MM cell lines (Oerlemans et al., 2008). However, the same mutations have not been confirmed in patients with MM resistant to bortezomib treatment. Bortezomib-resistant MM cells also display transcriptome alterations including increased antiapoptotic protein and decreased proapoptotic protein expression (Zhu et al., 2011). For example, bortezomib-resistant MM cells have higher Bcl-2 family (Smith et al., 2011) and heat shock protein (Hsp27, Hsp70, and Hsp90) expression, which is expected to mitigate the misfolded protein burden in these cells.

Other extrinsic factors contribute to bortezomib resistance. Not surprisingly, the bone marrow microenvironment plays an important role in supporting bortezomib resistance. Bone marrow stem cells (BMSCs) isolated from patients with bortezomib-resistance MM have different cytokine profiles than BMSCs from patients with bortezomib-sensitive MM (Hideshima et al., 2005). Several specific prosurvival cytokines and microRNAs are upregulated and contribute to bortezomib resistance (Hao et al., 2011). Interestingly, BMSCs from bortezomib-resistant patients confer resistance to proteasome inhibitor–naïve MM cells, whereas proteasome inhibitor–naïve MM cells cocultured with BSMCs from patients with bortezomib-sensitive MM respond to subsequent bortezomib exposure (Hao et al., 2011). The elucidation of specific microenvironment mechanisms supporting bortezomib resistance is expected to identify additional targets for combination therapies to enhance proteasome inhibitor sensitivity in patients with RRMM.

#### 3. Bortezomib Dose-Limiting Toxicity

The primary dose-limiting bortezomib treatment toxicity is peripheral neuropathy (PN). Bortezomib-induced PN typically affects the peripheral nervous system (PNS) sensory fibers and is associated with a painful burning sensation, numbness, and/or tingling in the extremities. Clinical trials report a bortezomib-induced PN incidence (all grades) between 30% and 60% (Kerckhove et al., 2017), with grade ≥3 occurring between 2% and 23% (Schlafer et al., 2017). PN is a major cause of dose reduction or discontinuation among patients and overcoming this limitation is a significant challenge to clinicians and pharmaceutical developers. Recent clinical trials demonstrated that patients receiving alternative dosing schedules (i.e., once weekly, instead of biweekly) or subcutaneous injection of bortezomib (instead of intravenously) had a lower incidence of PN, without changes in efficacy. For patients with intolerable PN, these options constitute another avenue of hope before discontinuing treatment (Schlafer et al., 2017).

Although the exact molecular mechanisms by which bortezomib induces PN are not completely clear, clinical and experimental evidence points to pathology in the primary sensory neuron cell bodies as a major contributing factor. The PNS encompasses the nerve fibers and cell bodies that reside outside the central nervous system (CNS) (i.e., the brain and spinal cord). Sensory receptors in periphery tissues transduce physical stimuli (e.g., pain, touch, pressure, temperature) into action potentials, which are transmitted via primary sensory neurons to the CNS. The primary sensory neuron cell bodies are in the dorsal root ganglion (DRG) just outside of the spinal cord. Many in vivo mouse studies and in vitro DRG explant studies of bortezomib-induced PN demonstrate accumulation of ubiquitinated proteins in DRG soma, defects in mitochondrial calcium homeostasis, disrupted mitochondrial axonal transport, alterations in tubulin polymerization and localization, and defects in fast axonal transport due to blockage of axonal protein turnover (Carozzi et al., 2010; Dasuri et al., 2010; Staff et al., 2013).

Why are the DRG neurons especially susceptible to proteasome inhibitor toxicity? Proper CNS and PNS neuron function requires an exquisitely controlled microenvironment. To this end, the blood-neural barrier and the blood-brain barrier form a protective barrier between the changing circulatory milieu and the PNS and CNS, respectively. These intricate barriers contain complex tight junction proteins. Unlike the rest of the nervous system, the cell body-rich area within the DRG has endothelial fenestrations and lacks tight junction proteins, rendering it more permeable to substances in the blood compared with the rest of the nervous system. This region is highly vascularized and blood permeability has been observed in human subjects using magnetic resonance imaging with gadolinium contrast agents (Kerckhove et al., 2017). Although bortezomib cannot cross the tight blood-neural barrier and blood-brain barrier, it can cross into the cell body-rich region of the DRG and inhibit proteasome function. This differential permeability is thought to underlie peripheral sensory system vulnerability to cytostatic agents used in chemotherapy (e.g., bortezomib) compared with other neurons in the CNS (Kerckhove et al., 2017).

Arastu-Kapur et al. (2011) suggested that bortezomib-induced neurotoxicity is due to mitochondrial serine protease, HtrA2/Omi, inhibition and independent of proteasome inhibition. They also showed that a second-generation proteasome inhibitor, carfilzomib, did not inhibit HtrA2/Omi in this study. The authors concluded that proteasome inhibitor–induced neurotoxicity is due to off-target bortezomib effects and is not generalizable to the proteasome inhibitor class. However, two subsequent independent studies failed to show bortezomib-mediated HtrA2 inhibition (Bachovchin et al., 2014; Csizmadia et al., 2016). Carfilzomib is associated with less severe PN than bortezomib; however, the role of HtrA2 or other off-target effects in proteasome inhibitor–induced PN remains to be determined.

#### 4. Bortezomib Efficacy in Solid Tumors

Despite bortezomib’s clinical efficacy in treating hematologic malignancies, it has had limited success in clinical trials for solid tumors. This may arise from poor bortezomib tissue penetration (at the doses used in MM) and rapid clearance from the blood (Adams, 2002). Due to an increased risk for PN, the dosage cannot be increased to overcome poor tissue penetration. Second-generation proteasome inhibitors are in development to overcome these limitations of bortezomib. Ongoing clinical trials are evaluating combinations of chemotherapy and radiotherapy with bortezomib in a search for new synergistic drug combinations.

### B. Second-Generation Proteasome Inhibitors

Bortezomib treatment efficacy in human cancer renewed interest in developing other novel proteasome inhibitors to overcome bortezomib limitations. The FDA has approved two second-generation proteasome inhibitors, carfilzomib (2012) and ixazomib (2015), for use in treating patients with RRMM. Carfilzomib and ixazomib are well tolerated in heavily pretreated patients and show effectiveness in bortezomib-resistant cases (Schlafer et al., 2017). Importantly, most cases of PN with carfilzomib and ixazomib are low grade and usually do not worsen preexisting PN resultant from previous bortezomib treatment (Schlafer et al., 2017). Schlafer et al. (2017) provide a detailed review of the bortezomib, carfilzomib, and ixazomib clinical trials. Here we highlight second-generation FDA-approved proteasome inhibitors and selected newer proteasome inhibitors undergoing preclinical evaluation in hematologic cancers, solid tumors, and autoimmune disorders. Our list of proteasome inhibitors is not intended to be comprehensive but rather to increase familiarity with these important aspects. Table 4 lists important properties of first- and second-generation proteasome inhibitors in clinical trials.

**TABLE 4:** Proteasome inhibitors in clinical testing

Inhibitor	Class	IC_50_ In Vitro Peptidase Activity			Half-Life	Route	Binding	Reference
		CT-L	C-L	T-L				
		*nM*			*min*			
Bortezomib	Boronate	7.9	53	590	110	i.v./s.c.	Slowly reversible	Cauhan et al. (2005)
Carfilzomib	Epoxyketone	6	2400	3600	<30	i.v.	Irreversible	Kuhn et al. (2007)
Ixazomib	Boronate	3.4	31	3500	18	p.o.	Reversible	Cauhan et al. (2005), Kupperman et al. (2010)
Marizomib	*β*-lactone	3.5	430	28	10–15	i.v.	Irreversible	Feling et al. (2003)
Oprozomib	Epoxyketone	36 (*β*5)	ND	ND	30–90	p.o.	Irreversible	Zhou et al. (2009), Roccaro et al. (2010)
		82 (*β*5i)						

C-L, caspase-like activity (*β*1); CT-L, chymotrypsin-like activity (*β*5); ND, not determined; T-L, trypsin-like activity (*β*2).

#### 1. Carfilzomib

Carfilzomib is the second FDA-approved proteasome inhibitor. Carfilzomib is used as a single agent or in combination with immunomodulatory agents for patients with RRMM who have received one to three prior therapies, including other proteasome inhibitors (Steele, 2013). Carfilzomib has a tripeptide backbone containing phenylalanine, leucine, and homophenylalanine with a terminal epoxyketone group that forms an irreversible covalently bond with the proteasome catalytic threonine (Sugumar et al., 2015). As with epoxomicin, the carfilzomib epoxyketone warhead forms a dual covalent adduct with the active site threonine, which greatly reduces the off targets.

Importantly, carfilzomib provides some therapeutic benefit for patients with RRMM who relapse after bortezomib treatment, while also rendering less neurotoxic side effects (Steele, 2013; Schlafer et al., 2017). The phase III ENDEAVOR (A Randomized, Open-label, Phase 3 Study of Carfilzomib Plus Dexamethasone vs. Bortezomib Plus Dexamethasone in Patients With Relapsed Multiple Myeloma) clinical trial compared bortezomib plus dexamethasone versus carfilzomib plus dexamethasone in a cohort of patients with newly diagnosed MM. ENDEAVOR reported a significantly higher incidence of ≥2 PN in the bortezomib group (32%) versus 6% in the carfilzomib group (Dimopoulos et al., 2017). This was the first head-to-head comparison between bortezomib- and carfilzomib-treated patients. Unfortunately, drug resistance is observed after carfilzomib treatment in a small subset of patients (Buac et al., 2013). Although the resistance mechanism is not fully elucidated, studies suggest that increased expression of drug efflux pump P-glycoprotein (a known transporter of carfilzomib) in carfilzomib-resistant cells contributes to the resistant phenotype (Ao et al., 2012). However, more detailed studies are needed to confirm this resistance mechanism.

Carfilzomib also shows therapeutic promise in several preclinical models of solid tumors. Carfilzomib effectively sensitized tumor cells to doxorubicin-induced apoptosis in several in vivo preclinical solid tumor models, including neuroblastoma and colon cancer (Li et al., 2016; Fan and Liu, 2017). Many doxorubicin-resistant tumor cells have upregulated NF-*κ*B activity that promotes survival (Enzler et al., 2011; Esparza-López et al., 2013), suggesting that combination treatment with a proteasome inhibitor may be effective at overcoming doxorubicin resistance. Although the exact mechanisms are unknown, it is thought that carfilzomib-mediated NF-*κ*B inhibition sensitizes tumor cells to doxorubicin. Based on these preclinical studies, carfilzomib (in combination with other chemotherapy drugs) is currently undergoing phase I and II clinical trials in advanced solid tumors, renal disease, transplant rejection, and hematologic malignancies (ClinicalTrials.gov).

#### *2. Ixazomib*

Bortezomib and carfilzomib are administered intravenously, requiring patients to visit a clinic several times over the course of their treatment. Ixazomib (MLN9708 [4-(carboxymethyl)-2-[(1*R*)-1-[[2-[(2,5-dichlorobenzoyl)amino]acetyl]amino]-3-methylbutyl]-6-oxo-1,3,2-dioxaborinane-4-carboxylic acid]) (Ninlaro; Takeda Pharmaceuticals), is the first and currently only orally bioavailable proteasome inhibitor approved by the FDA for RRMM treatment (Moreau et al., 2016). Ixazomib was developed through a large-scale, boron-containing proteasome inhibitor screening for compounds with physiochemical properties distinct from bortezomib. Ixazomib is a bioavailable prodrug and hydrolyses into the active metabolite MLN2238 ([(1*R*)-1-[[2-[(2,5-dichlorobenzoyl)amino]acetyl]amino]-3-methylbutyl]boronic acid) when exposed to the gastrointestinal tract and plasma and is a potent and reversible *β*5 proteasome subunit inhibitor (Muz et al., 2016).

Despite similarities, there are important distinctions between ixazomib and bortezomib. Importantly, clinical trials report a lower incidence of PN in patients treated with ixazomib compared with bortezomib, and ixazomib is effective in treating patients with bortezomib-resistant RRMM (Mateos et al., 2017). In addition, ixazomib demonstrated five times higher drug distribution supported by blood volume distribution, with a blood volume distribution of 20.2 l/kg for ixazomib versus 4.3 l/kg for bortezomib (Muz et al., 2016). Ixazomib also displays antitumor efficacy in solid tumor preclinical models (Fan and Liu, 2017). Numerous phase I and II clinical trials are underway, evaluating ixazomib treatment in glioblastomas (GBMs), solid tumors, triple-negative breast cancer, B-cell lymphoma, lupus, metastatic bladder cancer, and lymphoma (ClinicalTrials.gov).

### C. Additional Proteasome Inhibitors in Clinical Trials

#### 1. Marizomib

Marizomib (NPI-0052 [(1*S*,2*R*,5*R*)-2-(2-chloroethyl)-5-[(*S*)-[(1*S*)-cyclohex-2-en-1-yl]-hydroxymethyl]-1-methyl-7-oxa-4-azabicyclo[3.2.0]heptane-3,6-dione]; Salinosporamide A), is a naturally occurring proteasome inhibitor isolated from the marine actinomycete *Salinispora tropica* and is under development for MM and GBM treatment (Dou and Zonder, 2014). Marizomib is a *γ*-lactam–*β*-lactone that demonstrates unique binding and bioavailability profiles, setting it apart from other proteasome inhibitors. Its unusual binding mechanism was elucidated using biochemical and crystallography approaches. In contrast to other *β*-lactones, marizomib has unique chloroethyl and cyclohex-2-enylcarbinol substituents, giving rise to important interactions within proteasome active sites. These unique interactions are thought to be responsible for marizomib’s high proteasome affinity and specificity (Groll and Huber, 2004).

Marizomib irreversibly inhibits *β*5 (IC_50_ of 3.5 nM) and *β*2 (IC_50_ of 28 nM) active sites, although it can inhibit *β*1 at higher concentrations (IC_50_ of 430 nM) (Chauhan et al., 2005). In vitro and binding competition experiments show that marizomib binds to all three subunits at clinically relevant doses (Chauhan et al.2005). Unexpectedly, patients’ *β*1 and *β*2 activities were not affected (in PBMCs) during the first cycle of marizomib treatment (Levin et al., 2016). After the second dose cycle, decreased peptidase was observed but only in packed whole blood samples, which contain mostly erythrocytes (Levin et al., 2016). Whether marizomib inhibits *β*1 and *β*2 activity in nucleated cells that can synthesize new proteins remains to be determined. Importantly, marizomib shows the ability to overcome bortezomib and carfilzomib resistance in a limited number of patients with RRMM (Levin et al., 2016). Additional trials investigating marizomib in combination with other chemotherapy drugs in RRMM are ongoing.

Marizomib is currently the only proteasome inhibitor in clinical trials that can permeate the blood-brain barrier, making it an attractive candidate in CNS malignancy treatment. In animal studies, marizomib distributed to the brain at 30% blood levels in rats and significantly inhibited (>30%) baseline chymotrypsin-like proteasome activity in monkey brain tissue (Di et al., 2016). Furthermore, marizomib treatment elicited a significant antitumor effect in a rodent intracranial malignant glioma model (Di et al., 2016) and was well tolerated in phase I/II clinical trials for advanced and newly diagnosed GBM. Based on encouraging results from phase I and phase II marizomib trials in patients with GBM, a phase III trial of marizomib in combination with standard temozolomide-based radiochemotherapy for patients with newly diagnosed GBM (ClinicalTrials.gov NCT03345095) was scheduled to begin June 2018. GBM is the most common high-grade brain malignancy in adults, with a 25% 2-year survival after standard treatment (van Tellingen et al., 2015). New therapies are critically needed for these patients.

#### 2. Oprozomib

Efforts to synthesize an orally bioavailable epoxyketone proteasome inhibitor led to the development of oprozomib [ONX-0912 or PR-047 (*N*-[(2*S*)-3-methoxy-1-[[(2*S*)-3-methoxy-1-[[(2*S*)-1-[(2*R*)-2-methyloxiran-2-yl]-1-oxo-3-phenylpropan-2-yl]amino]-1-oxopropan-2-yl]amino]-1-oxopropan-2-yl]-2-methyl-1,3-thiazole-5-carboxamide)]. Oprozomib is an irreversible and potent *β*5c and *β*5i proteasome subunit inhibitor, with IC_50_ values of 36 and 82 nM, respectively (Laubach et al., 2009). Oprozomib oral administration demonstrated antitumor activity in MM, squamous cell carcinoma of the head and neck, and colorectal cancer preclinical models (Roccaro et al., 2010)

The first phase I study (ClinicalTrials.gov NCT01365559) with single-agent oprozomib evaluated patients with advanced solid tumors. Oprozomib was well tolerated, with low-grade gastrointestinal side effects being the most common. Unfortunately, despite dose-dependent increases in proteasome inhibition, the best response achieved was stable disease (23% of patients) and no clinically meaningful correlates between proteasome inhibition and treatment efficacy were observed. Considering that this was a heavily pretreated patient population with advanced cancer, the lack of a therapeutic response does not necessarily reflect the potential of oprozomib to treat earlier stages or other cancers. Clinical studies investigating the efficacy of oprozomib in hematologic malignancies are ongoing. A new oral oprozomib formulation is being investigated in a phase I/II study (ClinicalTrials.gov NCT01416428), and other studies are investigating oprozomib in combination with dexamethasone and lenalidomide.

### D. Immunoproteasome-Specific Proteasome Inhibitors

Specifically targeting the immunoproteasome could be advantageous over currently approved proteasome inhibitors in treating certain diseases. The immunoproteasome is present at low levels in normal cells. In contrast, some cancers (including MM), inflammatory diseases, and autoimmune diseases have increased immunoproteasome subunit expression (Kuhn et al., 2011). It is thought that selective immunoproteasome inhibition may have less adverse effects than broadly acting proteasome inhibitors like bortezomib and carfilzomib, since certain cell populations would be preferentially affected (Basler et al., 2015).

ONX-0914 (or PR-957 [(2*S*)-3-(4-methoxyphenyl)-*N*-[(2*S*)-1-[(2*R*)-2-methyloxiran-2-yl]-1-oxo-3-phenylpropan-2-yl]-2-[[(2*S*)-2-[(2-morpholin-4-ylacetyl)amino]propanoyl]amino]propanamide]) is an irreversible epoxyketone *β*5i immunoproteasome subunit inhibitor, with minimal crossreactivity for the constitutive proteasome *β*5 subunit (*β*5c). Preclinical trials demonstrate that *β*5i inhibition with ONX-0914 attenuates disease progression in colorectal cancer (Liu et al., 2017), rheumatoid arthritis (Muchamuel et al., 2009), and systemic lupus erythematosus animal models (Ichikawa et al., 2012). Importantly, ONX-0914 displays low neurotoxicity without sacrificing efficacy, an effect that may be attributed to the selective *β*5i inhibition (von Brzezinski et al., 2017).

Johnson et al. (2017) synthesized and screened ONX-0914 analogs for *β*2i inhibition. They identified and characterized compound KZR-504 (*N*-[(2*S*)-3-hydroxy-1-[[(2*S*)-1-[(2*R*)-2-methyloxiran-2-yl]-1-oxo-3-phenylpropan-2-yl]amino]-1-oxopropan-2-yl]-6-oxo-1*H*-pyridine-2-carboxamide), a *β*2i-selective proteasome inhibitor. However, KZR-504 displayed poor membrane permeability and did not ameliorate cytokine release from stimulated splenocytes (Johnson et al., 2017).

Another analog, KZR-616 [(2*S*,3*R*)-*N*-((*S*)-3-(cyclopent-1-en-1-yl)-1-((*R*)-2-methyloxiran-2-yl)-1-oxopropan-2-yl)-3-hydroxy-3-(4-methoxyphenyl)-2-((*S*)-2-(2-morpholinoacetamido)propanamido)propanamide], was well tolerated in a phase I clinical trial in healthy volunteers with minimal adverse side effects (Lickliter et al., 2017). KZR‐616 recently entered a phase Ib/II clinical trial (ClinicalTrials.gov NCT03393013) as a single-agent treatment of autoimmune‐triggered inflammation (systemic lupus erythematosus).

### E. Novel Combination Therapies

Anticancer drugs are often administered in combination to synergistically enhance cytotoxicity and prevent drug-resistant tumor cell population development. Several known chemotherapy resistance mechanisms result from abrogated proteasome activity. For example, NF-*κ*B activity is upregulated in solid tumors that develop chemotherapy (e.g., doxorubicin) or radiation resistance and is a driving force behind the resistant phenotype (Kisselev and Goldberg, 2001). Accordingly, preclinical studies with solid-tumor xenograft and cellular models show increased sensitivity to chemotherapy and/or radiation-induced apoptosis when combined with proteasome inhibitors (Holbeck et al., 2017). Several phase I and II clinical trials are evaluating proteasome inhibitor safety and efficacy in combination with various chemotherapy agents in recurrent or refractory advanced cancers.

*β*1 and *β*2 site upregulation has been reported in tumor cells resistant to bortezomib or carfilzomib treatment (Kisselev and Goldberg, 2001). LU-102 (*N*-[1-[4-(aminomethyl)-3-methylsulfonylphenyl]but-3-en-2-yl]-5-[[(2*S*)-2-azido-3-phenylpropanoyl]amino]-2-(2-methylpropyl)-4-oxooctanamide) is a peptide vinyl-sulfone *β*2-specific inhibitor (Kisselev et al., 2012) and alone it is toxic to MM cells, but it also synergized with *β*5 inhibitors (bortezomib and carfilzomib) to overcome proteasome inhibitor resistance in MM cell lines (Kisselev et al., 2012). The observed synergy between *β*5 and *β*2 inhibitors suggests that multiple site-specific proteasome inhibitor combinations may be an effective alternative in proteasome inhibitor–resistant malignancies with a reduced risk of adverse side effects.

## VI. Novel Proteasome Drug Targets

### A. Deubiquitinating Enzyme Inhibitors

With three ubiquitin receptors on the proteasome, several shuttling factors, and multiple ubiquitin chain types on substrates, the process of substrate recognition by the 26S proteasome is incredibly complex and much remains unknown. We briefly summarized the process of ubiquitin-degradation in section II; here we discuss how DUBs may be targeted to treat diseases. Detailed reviews of 26S substrate recognition and processing have already been published (Collins and Goldberg, 2017; de Poot et al., 2017).

Three DUBs are associated with the 26S proteasome: Rpn11, Uch37, and Usp14. Rpn11 is the only DUB that is an integral part of the 19S regulatory particle. Positioned directly above the translocation pore, Rpn11 is a metalloprotease that removes ubiquitin chains at their attachment point (lysine) on the substrate that is committed for degradation (Matyskiela et al., 2013). Mutations in Rpn11 that disrupt its catalytic activity stall ubiquitin substrate degradation and eventually lead to cell death (Verma et al., 2002). Li et al. (2017) recently developed capzimin, a first-in-class selective inhibitor of Rpn11. The Rpn11 active site is located within the highly conserved JAMM motif and features a catalytic Zn^2+^ ion (Verma et al., 2002). Capzimin binds the catalytic zinc ion and prevents Rpn11 activity (Li et al., 2017). Remarkably, capzimin treatment stabilized proteasome substrates and blocked proliferation in several tumor cell lines (Li et al., 2017). This antitumor activity suggests that Rpn11 inhibition may be an effective alternative to active site inhibition for treating malignancies.

Unlike Rpn11, Uch37 and Usp14 are only transiently associated with the 19S regulatory particle (Borodovsky et al., 2001; Leggett et al., 2002). Uch37 and Usp14 enzymes trim ubiquitin chains before the substrate is committed to degradation, which may suppress protein degradation by promoting early substrate release (de Poot et al., 2017). Accordingly, loss of Usp14 homolog Ubp6 increases substrate degradation by the proteasome (Lee et al., 2010; Kim and Goldberg, 2017). Despite high affinity, Usp14 easily dissociates from 26S complexes during conventional proteasome purification techniques and should be considered when designing in vitro experiments (de Poot et al., 2017).

Enhancing proteasome function has the potential to treat protein misfolding disorders, such as neurodegenerative diseases, and Usp14 inhibition may promote ubiquitin-dependent protein degradation. To this end, Boselli et al. (2017) developed IU-47, a potent and specific inhibitor of Usp14. Inhibition of Usp14 DUB activity by IU-47 promoted proteasome degradation of the microtubule-associated protein *τ* (implicated in the pathogenesis of Alzheimer disease) in neuronal cultures and enhanced resistance to oxidative stress (Boselli et al., 2017). The implications of pharmacological proteasome activation are discussed in the following section.

### B. Activation of 20S by Gate Opening

We highlighted essential functions of the proteasome required for proper neuronal functioning in section II. Then section III discussed the negative impacts of proteasome inhibition on primary sensory neurons. To bridge these concepts, we will further discuss evidence that supports the proteasome as a target for pharmacological activation to treat proteopathies.

The most common neurodegenerative diseases are characterized by an accumulation of aggregation-prone proteins concomitant with a loss of proteostasis, which results in progressive death of neurons (Selkoe, 2003; Rubinsztein, 2006; Brettschneider et al., 2015). Impaired proteasome function has been implicated, as a primary cause or a secondary consequence, in the pathogenesis of many neurodegenerative diseases, including Alzheimer, Parkinson, and Huntington diseases (Keller et al., 2000; McNaught et al., 2001; Ciechanover and Brundin, 2003; Ortega et al., 2007; McKinnon and Tabrizi, 2014).

Soluble forms of aggregated proteins, called oligomers, are implicated in the pathogenesis of most neurodegenerative diseases (Haass and Selkoe, 2007; Guerrero-Muñoz et al., 2014) and haven been shown to impair proteasome function (Bence et al., 2001; Lindersson et al., 2004; Díaz-Hernández et al., 2006; Cecarini et al., 2008; Tseng et al., 2008; Deriziotis et al., 2011). Our laboratory recently identified a mechanism in which soluble oligomers of different proteins from multiple neurodegenerative diseases allosterically impair the proteasome gate by a shared mechanism (Thibaudeau et al., 2018). These toxic oligomers shared a similar three-dimensional conformation recognized by the antioligomer antibody, A11 (Kayed et al., 2003). The A11+ oligomers bound to the outside of the 20S cylinder and stabilized the gate in the closed conformation. However, proteasome function could be rescued by adding eight-residue HbYX peptides. The HbYX peptides allosterically opened the gate and the HbYX motif peptides overcame the oligomer inhibition and restored proteasome function (Thibaudeau et al., 2018).

A recent and popular idea for treating neurodegenerative diseases is to enhance proteasome activity to suppress toxicity and related proteotoxic pathophysiology, but to date no drugs that directly activate proteasome function are available.

Since IU-47 exerts its effect upstream of the proteasome gate, it is not expected to function in conditions in which the proteasome gate is impaired. Choi et al. (2016) generated an HEK293 cell line with a stable transfection of a mutant 20S *α* subunit (*α*3∆N) that induces 20S gate opening. They showed that HEK293-*α*3∆N cells had increased degradation of proteasome substrates (including tau protein) and increased resistance to oxidative stress compared with the wild type (Choi et al., 2016). Although this serves as a proof of principle that opening the proteasome gate can clear aggregation-prone proteins and increase cell viability, it cannot be determined whether pharmacologically activating the proteasome in an already diseased state (preexisting oligomers, aggregates, and oxidative stress) can restore cellular proteostasis. Nonetheless, the elucidation of a common mechanism of proteasome inhibition is a major step toward the rational design of proteasome-activating compounds, which may be a promising route to restore proteostasis in diseases.

## VII. Conclusions

As a highly regulated, multicatalytic macromolecular complex, the proteasome possesses multiple drug targets to modulate its degradation capacity. Proteasome inhibitors helped early researchers study the proteasome’s cellular functions. Today, three inhibitors are approved for use in the clinic to treat hematologic cancers and several more inhibitors (synthetic and naturally occurring) are in preclinical and clinical testing. Fine-tuning the pharmacological properties of proteasome inhibitors may improve their efficacy for use in solid tumors. The last decade has witnessed major progress in our understanding of 26S proteasome structure and dynamics. In the coming years, we expect development of new inhibitors and activators of nonproteolytic components of the 26S proteasome. We anticipate that proteasome activation will become a validated target to treat proteotoxic diseases.

## Abbreviations

ABP: activity-based probe
amc: amino‐4‐methylcoumarin
AsnEDA: asparagine-ethylenediamine
BMSC: bone marrow stem cell
CNS: central nervous system
DRG: dorsal root ganglion
DUB: deubiquitinase
E-64: (2*S*,3*S*)-3-[[(2*S*)-1-[4-(diaminomethylideneamino)butylamino]-4-methyl-1-oxopentan-2-yl]carbamoyl]oxirane-2-carboxylic acid
EM: electron microscopy
ER: endoplasmic reticulum
ERAD: endoplasmic reticulum–associated protein degradation
FDA: U.S. Food and Drug Administration
GBM: glioblastoma
GFP: green fluorescent protein
GST: glutathione *S*-transferase
HbYX: hydrophobic-tyrosine-any residue
HEK293: human embryonic kidney 293
IFN: interferon
IKK: I*κ*B kinase
KZR-616: (2*S*,3*R*)-*N*-((*S*)-3-(cyclopent-1-en-1-yl)-1-((*R*)-2-methyloxiran-2-yl)-1-oxopropan-2-yl)-3-hydroxy-3-(4-methoxyphenyl)-2-((*S*)-2-(2-morpholinoacetamido)propanamido)propanamide
LU-102: *N*-[1-[4-(aminomethyl)-3-methylsulfonylphenyl]but-3-en-2-yl]-5-[[(2*S*)-2-azido-3-phenylpropanoyl]amino]-2-(2-methylpropyl)-4-oxooctanamide
MG-132: carbobenzyl-Leu-Leu-Leu-aldehyde
MHC-I: major histocompatibility complex class I
MLN2238 ([(1*R*)-1-[[2-[(2: 5-dichlorobenzoyl)amino]acetyl]amino]-3-methylbutyl]boronic acid
MLN9708: 4-(carboxymethyl)-2-[(1*R*)-1-[[2-[(2,5-dichlorobenzoyl)amino]acetyl]amino]-3-methylbutyl]-6-oxo-1,3,2-dioxaborinane-4-carboxylic acid
MM: multiple myeloma
NF-*κ*B: nuclear factor-*κ*B
NPI-0052: (1*S*,2*R*,5*R*)-2-(2-chloroethyl)-5-[(*S*)-[(1*S*)-cyclohex-2-en-1-yl]-hydroxymethyl]-1-methyl-7-oxa-4-azabicyclo[3.2.0]heptane-3,6-dione
ODC: ornithine decarboxylase
ONX-0912 (or PR-047): *N*-[(2*S*)-3-methoxy-1-[[(2*S*)-3-methoxy-1-[[(2*S*)-1-[(2*R*)-2-methyloxiran-2-yl]-1-oxo-3-phenylpropan-2-yl]amino]-1-oxopropan-2-yl]amino]-1-oxopropan-2-yl]-2-methyl-1,3-thiazole-5-carboxamide
ONX-0914 (or PR-957): (2*S*)-3-(4-methoxyphenyl)-*N*-[(2*S*)-1-[(2*R*)-2-methyloxiran-2-yl]-1-oxo-3-phenylpropan-2-yl]-2-[[(2*S*)-2-[(2-morpholin-4-ylacetyl)amino]propanoyl]amino]propanamide
PN: peripheral neuropathy
PNS: peripheral nervous system
PS-341: Pyz-Phe-boroLeu
RRMM: relapsed/refractory multiple myeloma
Suc-LLVY: succinyl-leucine-leucine-valine-tyrosine
TPP-II: tripeptidyl-protease II
UBL: ubiquitin-like
UFD: ubiquitin fusion degradation pathway
UPR: unfolded protein response
UPS: ubiquitin proteasome system

## References

[B1] Adams GM Crotchett B Slaughter CA DeMartino GN Gogol EP (1998 a) Formation of proteasome-PA700 complexes directly correlates with activation of peptidase activity. Biochemistry 37:12927–12932. 9737872 10.1021/bi981482i

[B2] Adams J (2001) Proteasome inhibition in cancer: development of PS-341. Semin Oncol 28:613–619. 11740819 10.1016/s0093-7754(01)90034-x

[B3] Adams J (2002) Proteasome inhibitors as new anticancer drugs. Curr Opin Oncol 14:628–634. 12409653 10.1097/00001622-200211000-00007

[B4] Adams J (2003) Potential for proteasome inhibition in the treatment of cancer. Drug Discov Today 8:307–315. 12654543 10.1016/s1359-6446(03)02647-3

[B5] Adams J Behnke M Chen S Cruickshank AA Dick LR Grenier L Klunder JM Ma YT Plamondon L Stein RL (1998 b) Potent and selective inhibitors of the proteasome: dipeptidyl boronic acids. Bioorg Med Chem Lett 8:333–338. 9871680 10.1016/s0960-894x(98)00029-8

[B6] Adams J Palombella VJ Sausville EA Johnson J Destree A Lazarus DD Maas J Pien CS Prakash S Elliott PJ (1999) Proteasome inhibitors: a novel class of potent and effective antitumor agents. Cancer Res 59:2615–2622. 10363983

[B7] An B Goldfarb RH Siman R Dou QP (1998) Novel dipeptidyl proteasome inhibitors overcome Bcl-2 protective function and selectively accumulate the cyclin-dependent kinase inhibitor p27 and induce apoptosis in transformed, but not normal, human fibroblasts. Cell Death Differ 5:1062–1075. 9894613 10.1038/sj.cdd.4400436

[B8] Ao L Wu Y Kim D Jang ER Kim K Lee DM Kim KB Lee W (2012) Development of peptide-based reversing agents for P-glycoprotein-mediated resistance to carfilzomib. Mol Pharm 9:2197–2205. 22734651 10.1021/mp300044b PMC3473138

[B9] Appelmans F Wattiaux R De Duve C (1955) Tissue fractionation studies. 5. The association of acid phosphatase with a special class of cytoplasmic granules in rat liver. Biochem J 59:438–445. 14363114 10.1042/bj0590438 PMC1216263

[B10] Arastu-Kapur S Anderl JL Kraus M Parlati F Shenk KD Lee SJ Muchamuel T Bennett MK Driessen C Ball AJ (2011) Nonproteasomal targets of the proteasome inhibitors bortezomib and carfilzomib: a link to clinical adverse events. Clin Cancer Res 17:2734–2743. 21364033 10.1158/1078-0432.CCR-10-1950

[B11] Arendt CS Hochstrasser M (1997) Identification of the yeast 20S proteasome catalytic centers and subunit interactions required for active-site formation. Proc Natl Acad Sci USA 94:7156–7161. 9207060 10.1073/pnas.94.14.7156 PMC23776

[B12] Arrigo AP Tanaka K Goldberg AL Welch WJ (1988) Identity of the 19S ‘prosome’ particle with the large multifunctional protease complex of mammalian cells (the proteasome). Nature 331:192–194. 3277060 10.1038/331192a0

[B13] Aso E Lomoio S López-González I Joda L Carmona M Fernández-Yagüe N Moreno J Juvés S Pujol A Pamplona R (2012) Amyloid generation and dysfunctional immunoproteasome activation with disease progression in animal model of familial Alzheimer’s disease. Brain Pathol 22:636–653. 22188425 10.1111/j.1750-3639.2011.00560.x PMC8057644

[B14] Bachovchin DA Koblan LW Wu W Liu Y Li Y Zhao P Woznica I Shu Y Lai JH Poplawski SE (2014) A high-throughput, multiplexed assay for superfamily-wide profiling of enzyme activity. Nat Chem Biol 10:656–663. 24997602 10.1038/nchembio.1578 PMC5953424

[B15] Baldin V Militello M Thomas Y Doucet C Fic W Boireau S Jariel-Encontre I Piechaczyk M Bertrand E Tazi J (2008) A novel role for PA28gamma-proteasome in nuclear speckle organization and SR protein trafficking. Mol Biol Cell 19:1706–1716. 18256291 10.1091/mbc.E07-07-0637 PMC2291414

[B16] Baldwin AS (2001) Control of oncogenesis and cancer therapy resistance by the transcription factor NF-kappaB. J Clin Invest 107:241–246. 11160144 10.1172/JCI11991 PMC199203

[B17] Bashore C Dambacher CM Goodall EA Matyskiela ME Lander GC Martin A (2015) Ubp6 deubiquitinase controls conformational dynamics and substrate degradation of the 26S proteasome. Nat Struct Mol Biol 22:712–719. 26301997 10.1038/nsmb.3075 PMC4560640

[B18] Basler M Mundt S Bitzer A Schmidt C Groettrup M (2015) The immunoproteasome: a novel drug target for autoimmune diseases. Clin Exp Rheumatol 33 (Suppl 92):S74–S79. 26458097

[B19] Bassermann F Eichner R Pagano M (2014) The ubiquitin proteasome system - implications for cell cycle control and the targeted treatment of cancer. Biochim Biophys Acta 1843:150–162. 23466868 10.1016/j.bbamcr.2013.02.028 PMC3694769

[B20] Bays NW Wilhovsky SK Goradia A Hodgkiss-Harlow K Hampton RY (2001) HRD4/NPL4 is required for the proteasomal processing of ubiquitinated ER proteins. Mol Biol Cell 12:4114–4128. 11739805 10.1091/mbc.12.12.4114 PMC60780

[B21] Beck F Unverdorben P Bohn S Schweitzer A Pfeifer G Sakata E Nickell S Plitzko JM Villa E Baumeister W (2012) Near-atomic resolution structural model of the yeast 26S proteasome. Proc Natl Acad Sci USA 109:14870–14875. 22927375 10.1073/pnas.1213333109 PMC3443124

[B22] Beckwith R Estrin E Worden EJ Martin A (2013) Reconstitution of the 26S proteasome reveals functional asymmetries in its AAA+ unfoldase. Nat Struct Mol Biol 20:1164–1172. 24013205 10.1038/nsmb.2659 PMC3869383

[B23] Bedford L Hay D Devoy A Paine S Powe DG Seth R Gray T Topham I Fone K Rezvani N (2008) Depletion of 26S proteasomes in mouse brain neurons causes neurodegeneration and Lewy-like inclusions resembling human pale bodies. J Neurosci 28:8189–8198. 18701681 10.1523/JNEUROSCI.2218-08.2008 PMC6670564

[B24] Ben-Neriah Y (2002) Regulatory functions of ubiquitination in the immune system. Nat Immunol 3:20–26. 11753406 10.1038/ni0102-20

[B25] Bence NF Bennett EJ Kopito RR (2005) Application and analysis of the GFPu family of ubiquitin-proteasome system reporters. Methods Enzymol 399:481–490. 16338377 10.1016/S0076-6879(05)99033-2

[B26] Bence NF Sampat RM Kopito RR (2001) Impairment of the ubiquitin-proteasome system by protein aggregation. Science 292:1552–1555. 11375494 10.1126/science.292.5521.1552

[B27] Bennett EJ Bence NF Jayakumar R Kopito RR (2005) Global impairment of the ubiquitin-proteasome system by nuclear or cytoplasmic protein aggregates precedes inclusion body formation. Mol Cell 17:351–365. 15694337 10.1016/j.molcel.2004.12.021

[B28] Besche HC Goldberg AL (2012) Affinity purification of mammalian 26S proteasomes using an ubiquitin-like domain. Methods Mol Biol 832:423–432. 22350902 10.1007/978-1-61779-474-2_29

[B29] Bhattacharyya S Renn JP Yu H Marko JF Matouschek A (2016) An assay for 26S proteasome activity based on fluorescence anisotropy measurements of dye-labeled protein substrates. Anal Biochem 509:50–59. 27296635 10.1016/j.ab.2016.05.026 PMC4976823

[B30] Bigelow S Hough R Rechsteiner M (1981) The selective degradation of injected proteins occurs principally in the cytosol rather than in lysosomes. Cell 25:83–93. 7273138 10.1016/0092-8674(81)90233-6

[B31] Blickwedehl J Olejniczak S Cummings R Sarvaiya N Mantilla A Chanan-Khan A Pandita TK Schmidt M Thompson CB Bangia N (2012) The proteasome activator PA200 regulates tumor cell responsiveness to glutamine and resistance to ionizing radiation. Mol Cancer Res 10:937–944. 22550082 10.1158/1541-7786.MCR-11-0493-T PMC5261820

[B32] Borissenko L Groll M (2007) 20S proteasome and its inhibitors: crystallographic knowledge for drug development. Chem Rev 107:687–717. 17316053 10.1021/cr0502504

[B33] Borodovsky A Kessler BM Casagrande R Overkleeft HS Wilkinson KD Ploegh HL (2001) A novel active site-directed probe specific for deubiquitylating enzymes reveals proteasome association of USP14. EMBO J 20:5187–5196. 11566882 10.1093/emboj/20.18.5187 PMC125629

[B34] Boselli M Lee BH Robert J Prado MA Min SW Cheng C Silva MC Seong C Elsasser S Hatle KM (2017) An inhibitor of the proteasomal deubiquitinating enzyme USP14 induces tau elimination in cultured neurons. J Biol Chem 292:19209–19225. 28972160 10.1074/jbc.M117.815126 PMC5702663

[B35] Braun S Matuschewski K Rape M Thoms S Jentsch S (2002) Role of the ubiquitin-selective CDC48(UFD1/NPL4) chaperone (segregase) in ERAD of OLE1 and other substrates. EMBO J 21:615–621. 11847109 10.1093/emboj/21.4.615 PMC125867

[B36] Brettschneider J Del Tredici K Lee VM Trojanowski JQ (2015) Spreading of pathology in neurodegenerative diseases: a focus on human studies. Nat Rev Neurosci 16:109–120. 25588378 10.1038/nrn3887 PMC4312418

[B37] Buac D Shen M Schmitt S Kona FR Deshmukh R Zhang Z Neslund-Dudas C Mitra B Dou QP (2013) From bortezomib to other inhibitors of the proteasome and beyond. Curr Pharm Des 19:4025–4038. 23181572 10.2174/1381612811319220012 PMC3657018

[B38] Cadima-Couto I Freitas-Vieira A Nowarski R Britan-Rosich E Kotler M Goncalves J (2009) Ubiquitin-fusion as a strategy to modulate protein half-life: A3G antiviral activity revisited. Virology 393:286–294. 19717177 10.1016/j.virol.2009.07.031

[B39] Carozzi VA Canta A Oggioni N Sala B Chiorazzi A Meregalli C Bossi M Marmiroli P Cavaletti G (2010) Neurophysiological and neuropathological characterization of new murine models of chemotherapy-induced chronic peripheral neuropathies. Exp Neurol 226:301–309. 20832406 10.1016/j.expneurol.2010.09.004

[B40] Carvalho P Goder V Rapoport TA (2006) Distinct ubiquitin-ligase complexes define convergent pathways for the degradation of ER proteins. Cell 126:361–373. 16873066 10.1016/j.cell.2006.05.043

[B41] Cecarini V Bonfili L Amici M Angeletti M Keller JN Eleuteri AM (2008) Amyloid peptides in different assembly states and related effects on isolated and cellular proteasomes. Brain Res 1209:8–18. 18400214 10.1016/j.brainres.2008.03.003

[B264] Chauhan D Catley L Li G Podar K Hideshima T Velankar M Mitsiades C Mitsiades N Yasui H Letai A (2005) A novel orally active proteasome inhibitor induces apoptosis in multiple myeloma cells with mechanisms distinct from Bortezomib. Cancer Cell 8:407–419. 16286248 10.1016/j.ccr.2005.10.013

[B43] Chen C Edelstein LC Gélinas C (2000) The Rel/NF-kappaB family directly activates expression of the apoptosis inhibitor Bcl-x(L). Mol Cell Biol 20:2687–2695. 10733571 10.1128/mcb.20.8.2687-2695.2000 PMC85484

[B44] Chen S Wu J Lu Y Ma Y-B Lee B-H Yu Z Ouyang Q Finley DJ Kirschner MW Mao Y (2016) Structural basis for dynamic regulation of the human 26S proteasome. Proc Natl Acad Sci USA 113:12991–12996. 27791164 10.1073/pnas.1614614113 PMC5135334

[B45] Chen X Barton LF Chi Y Clurman BE Roberts JM (2007) Ubiquitin-independent degradation of cell-cycle inhibitors by the REGgamma proteasome. Mol Cell 26:843–852. 17588519 10.1016/j.molcel.2007.05.022 PMC2031223

[B46] Chen Z Hagler J Palombella VJ Melandri F Scherer D Ballard D Maniatis T (1995) Signal-induced site-specific phosphorylation targets I kappa B alpha to the ubiquitin-proteasome pathway. Genes Dev 9:1586–1597. 7628694 10.1101/gad.9.13.1586

[B47] Chhabra S (2017) Novel proteasome inhibitors and histone deacetylase inhibitors: progress in myeloma therapeutics. Pharmaceuticals (Basel) 10:E40. 10.3390/ph10020040 PMC5490397 28398261

[B48] Choi WH de Poot SAH Lee JH Kim JH Han DH Kim YK Finley D Lee MJ (2016) Open-gate mutants of the mammalian proteasome show enhanced ubiquitin-conjugate degradation. Nat Commun 7:10963. 26957043 10.1038/ncomms10963 PMC4786872

[B49] Chou TF Deshaies RJ (2011) Quantitative cell-based protein degradation assays to identify and classify drugs that target the ubiquitin-proteasome system. J Biol Chem 286:16546–16554. 21343295 10.1074/jbc.M110.215319 PMC3089497

[B50] Ciechanover A (2005) Proteolysis: from the lysosome to ubiquitin and the proteasome. Nat Rev Mol Cell Biol 6:79–87. 15688069 10.1038/nrm1552

[B51] Ciechanover A (2013) Intracellular protein degradation: from a vague idea through the lysosome and the ubiquitin-proteasome system and onto human diseases and drug targeting. Bioorg Med Chem 21:3400–3410. 23485445 10.1016/j.bmc.2013.01.056

[B52] Ciechanover A Brundin P (2003) The ubiquitin proteasome system in neurodegenerative diseases: sometimes the chicken, sometimes the egg. Neuron 40:427–446. 14556719 10.1016/s0896-6273(03)00606-8

[B53] Ciechanover A Elias S Heller H Hershko A (1982) “Covalent affinity” purification of ubiquitin-activating enzyme. J Biol Chem 257:2537–2542. 6277904

[B54] Ciechanover A Finley D Varshavsky A (1984) Ubiquitin dependence of selective protein degradation demonstrated in the mammalian cell cycle mutant ts85. Cell 37:57–66. 6327060 10.1016/0092-8674(84)90300-3

[B55] Ciechanover A Heller H Elias S Haas AL Hershko A (1980) ATP-dependent conjugation of reticulocyte proteins with the polypeptide required for protein degradation. Proc Natl Acad Sci USA 77:1365–1368. 6769112 10.1073/pnas.77.3.1365 PMC348495

[B56] Ciechanover A Hod Y Hershko A (1978) A heat-stable polypeptide component of an ATP-dependent proteolytic system from reticulocytes. Biochem Biophys Res Commun 81:1100–1105. 666810 10.1016/0006-291x(78)91249-4

[B57] Collins GA Goldberg AL (2017) The logic of the 26S proteasome. Cell 169:792–806. 28525752 10.1016/j.cell.2017.04.023 PMC5609836

[B58] Csizmadia V Hales P Tsu C Ma J Chen J Shah P Fleming P Senn JJ Kadambi VJ Dick L (2016) Proteasome inhibitors bortezomib and carfilzomib used for the treatment of multiple myeloma do not inhibit the serine protease HtrA2/Omi. Toxicol Res (Camb) 5:1619–1628. 30090462 10.1039/c6tx00220j PMC6062231

[B59] da Fonseca PC Morris EP (2015) Cryo-EM reveals the conformation of a substrate analogue in the human 20S proteasome core. Nat Commun 6:7573. 26133119 10.1038/ncomms8573 PMC4506541

[B60] Dantuma NP Lindsten K Glas R Jellne M Masucci MG (2000) Short-lived green fluorescent proteins for quantifying ubiquitin/proteasome-dependent proteolysis in living cells. Nat Biotechnol 18:538–543. 10802622 10.1038/75406

[B61] Dasuri K Ebenezer PJ Zhang L Fernandez-Kim SO Uranga RM Gavilán E Di Blasio A Keller JN (2010) Selective vulnerability of neurons to acute toxicity after proteasome inhibitor treatment: implications for oxidative stress and insolubility of newly synthesized proteins. Free Radic Biol Med 49:1290–1297. 20678570 10.1016/j.freeradbiomed.2010.07.014 PMC3175605

[B62] de Duve C Gianetto R Appelmans F Wattiaux R (1953) Enzymic content of the mitochondria fraction. Nature 172:1143–1144. 13111270 10.1038/1721143a0

[B63] de Duve C Pressman BC Gianetto R Wattiaux R Appelmans F (1955) Tissue fractionation studies. 6. Intracellular distribution patterns of enzymes in rat-liver tissue. Biochem J 60:604–617. 13249955 10.1042/bj0600604 PMC1216159

[B64] de Duve C Wattiaux R (1966) Functions of lysosomes. Annu Rev Physiol 28:435–492. 5322983 10.1146/annurev.ph.28.030166.002251

[B65] de Poot SAH Tian G Finley D (2017) Meddling with fate: the proteasomal deubiquitinating enzymes. J Mol Biol 429:3525–3545. 28988953 10.1016/j.jmb.2017.09.015 PMC5675770

[B66] Dechavanne V Vilbois F Glez L Antonsson B (2013) Purification and separation of the 20S immunoproteasome from the constitutive proteasome and identification of the subunits by LC-MS. Protein Expr Purif 87:100–110. 23147206 10.1016/j.pep.2012.10.009

[B67] DeLaBarre B Christianson JC Kopito RR Brunger AT (2006) Central pore residues mediate the p97/VCP activity required for ERAD. Mol Cell 22:451–462. 16713576 10.1016/j.molcel.2006.03.036

[B68] DeMartino GN Proske RJ Moomaw CR Strong AA Song X Hisamatsu H Tanaka K Slaughter CA (1996) Identification, purification, and characterization of a PA700-dependent activator of the proteasome. J Biol Chem 271:3112–3118. 8621709 10.1074/jbc.271.6.3112

[B69] DeMartino GN Slaughter CA (1999) The proteasome, a novel protease regulated by multiple mechanisms. J Biol Chem 274:22123–22126. 10428771 10.1074/jbc.274.32.22123

[B70] Deriziotis P André R Smith DM Goold R Kinghorn KJ Kristiansen M Nathan JA Rosenzweig R Krutauz D Glickman MH (2011) Misfolded PrP impairs the UPS by interaction with the 20S proteasome and inhibition of substrate entry. EMBO J 30:3065–3077. 21743439 10.1038/emboj.2011.224 PMC3160194

[B71] Di K Lloyd GK Abraham V MacLaren A Burrows FJ Desjardins A Trikha M Bota DA (2016) Marizomib activity as a single agent in malignant gliomas: ability to cross the blood-brain barrier. Neuro-oncol 18:840–848. 26681765 10.1093/neuonc/nov299 PMC4864261

[B72] Díaz-Hernández M Valera AG Morán MA Gómez-Ramos P Alvarez-Castelao B Castaño JG Hernández F Lucas JJ (2006) Inhibition of 26S proteasome activity by huntingtin filaments but not inclusion bodies isolated from mouse and human brain. J Neurochem 98:1585–1596. 16787406 10.1111/j.1471-4159.2006.03968.x

[B73] Dietrich C Bartsch T Schanz F Oesch F Wieser RJ (1996) p53-dependent cell cycle arrest induced by N-acetyl-L-leucinyl-L-leucinyl-L-norleucinal in platelet-derived growth factor-stimulated human fibroblasts. Proc Natl Acad Sci USA 93:10815–10819. 8855263 10.1073/pnas.93.20.10815 PMC38238

[B74] Dimopoulos MA Goldschmidt H Niesvizky R Joshua D Chng WJ Oriol A Orlowski RZ Ludwig H Facon T Hajek R (2017) Carfilzomib or bortezomib in relapsed or refractory multiple myeloma (ENDEAVOR): an interim overall survival analysis of an open-label, randomised, phase 3 trial. Lancet Oncol 18:1327–1337. 28843768 10.1016/S1470-2045(17)30578-8

[B75] Djakovic SN Marquez-Lona EM Jakawich SK Wright R Chu C Sutton MA Patrick GN (2012) Phosphorylation of Rpt6 regulates synaptic strength in hippocampal neurons. J Neurosci 32:5126–5131. 22496558 10.1523/JNEUROSCI.4427-11.2012 PMC3348785

[B76] Dohmen RJ Varshavsky A (2005) Heat-inducible degron and the making of conditional mutants. Methods Enzymol 399:799–822. 16338396 10.1016/S0076-6879(05)99052-6

[B77] Dou QP Smith DM Daniel KG Kazi A (2003) Interruption of tumor cell cycle progression through proteasome inhibition: implications for cancer therapy. Prog Cell Cycle Res 5:441–446. 14593738

[B78] Dou QP Zonder JA (2014) Overview of proteasome inhibitor-based anti-cancer therapies: perspective on bortezomib and second generation proteasome inhibitors versus future generation inhibitors of ubiquitin-proteasome system. Curr Cancer Drug Targets 14:517–536. 25092212 10.2174/1568009614666140804154511 PMC4279864

[B79] Dulić V Kaufmann WK Wilson SJ Tlsty TD Lees E Harper JW Elledge SJ Reed SI (1994) p53-dependent inhibition of cyclin-dependent kinase activities in human fibroblasts during radiation-induced G1 arrest. Cell 76:1013–1023. 8137420 10.1016/0092-8674(94)90379-4

[B80] Enzler T Sano Y Choo MK Cottam HB Karin M Tsao H Park JM (2011) Cell-selective inhibition of NF-κB signaling improves therapeutic index in a melanoma chemotherapy model. Cancer Discov 1:496–507. 22389871 10.1158/2159-8290.CD-11-0143 PMC3290412

[B81] Esparza-López J Medina-Franco H Escobar-Arriaga E León-Rodríguez E Zentella-Dehesa A Ibarra-Sánchez MJ (2013) Doxorubicin induces atypical NF-κB activation through c-Abl kinase activity in breast cancer cells. J Cancer Res Clin Oncol 139:1625–1635. 23892407 10.1007/s00432-013-1476-3 PMC11824581

[B82] Etlinger JD Goldberg AL (1977) A soluble ATP-dependent proteolytic system responsible for the degradation of abnormal proteins in reticulocytes. Proc Natl Acad Sci USA 74:54–58. 264694 10.1073/pnas.74.1.54 PMC393195

[B83] Falk K Rötzschke O (1993) Consensus motifs and peptide ligands of MHC class I molecules. Semin Immunol 5:81–94. 7684938 10.1006/smim.1993.1012

[B84] Fan Q Liu B (2017) Identification of the anticancer effects of a novel proteasome inhibitor, ixazomib, on colorectal cancer using a combined method of microarray and bioinformatics analysis. OncoTargets Ther 10:3591–3606. 10.2147/OTT.S139686 PMC5530849 28790851

[B265] Feling RH Buchanan GO Mincer TJ Kauffman CA Jensen PR Fenical W (2003) A highly cytotoxic proteasome inhibitor from a novel microbial source, a marine bacterium of the new genus salinospora. Angew Chem Int Ed Engl. 42:355–357. 12548698 10.1002/anie.200390115

[B85] Finley D Chen X Walters KJ (2016) Gates, channels, and switches: elements of the proteasome machine. Trends Biochem Sci 41:77–93. 26643069 10.1016/j.tibs.2015.10.009 PMC4706478

[B86] Finley D Ciechanover A Varshavsky A (1984) Thermolability of ubiquitin-activating enzyme from the mammalian cell cycle mutant ts85. Cell 37:43–55. 6327059 10.1016/0092-8674(84)90299-x

[B87] Fonseca R Vabulas RM Hartl FU Bonhoeffer T Nägerl UV (2006) A balance of protein synthesis and proteasome-dependent degradation determines the maintenance of LTP. Neuron 52:239–245. 17046687 10.1016/j.neuron.2006.08.015

[B88] Foresti O Rodriguez-Vaello V Funaya C Carvalho P (2014) Quality control of inner nuclear membrane proteins by the Asi complex. Science 346:751–755. 25236469 10.1126/science.1255638

[B89] Gaczynska M Rock KL Goldberg AL (1993) Gamma-interferon and expression of MHC genes regulate peptide hydrolysis by proteasomes. Nature 365:264–267. 8396732 10.1038/365264a0

[B90] Ghoda L van Daalen Wetters T Macrae M Ascherman D Coffino P (1989) Prevention of rapid intracellular degradation of ODC by a carboxyl-terminal truncation. Science 243:1493–1495. 2928784 10.1126/science.2928784

[B91] Gilon T Chomsky O Kulka RG (1998) Degradation signals for ubiquitin system proteolysis in Saccharomyces cerevisiae. EMBO J 17:2759–2766. 9582269 10.1093/emboj/17.10.2759 PMC1170616

[B92] Glickman MH Ciechanover A (2002) The ubiquitin-proteasome proteolytic pathway: destruction for the sake of construction. Physiol Rev 82:373–428. 11917093 10.1152/physrev.00027.2001

[B93] Goldberg AL (1972) Degradation of abnormal proteins in Escherichia coli (protein breakdown-protein structure-mistranslation-amino acid analogs-puromycin). Proc Natl Acad Sci USA 69:422–426. 4551144 10.1073/pnas.69.2.422 PMC426471

[B94] Goldberg AL (2007) Functions of the proteasome: from protein degradation and immune surveillance to cancer therapy. Biochem Soc Trans 35:12–17. 17212580 10.1042/BST0350012

[B95] Goldberg AL (2012) Development of proteasome inhibitors as research tools and cancer drugs. J Cell Biol 199:583–588. 23148232 10.1083/jcb.201210077 PMC3494858

[B96] Goldberg AL (2016) Alfred L. Goldberg: probing the proteasome. Trends Cell Biol 26:792–794. 27680754 10.1016/j.tcb.2016.09.003

[B97] Goldberg AL Dice JF (1974) Intracellular protein degradation in mammalian and bacterial cells. Annu Rev Biochem 43:835–869. 4604628 10.1146/annurev.bi.43.070174.004155

[B98] Goldberg AL St John AC (1976) Intracellular protein degradation in mammalian and bacterial cells: part 2. Annu Rev Biochem 45:747–803. 786161 10.1146/annurev.bi.45.070176.003531

[B99] Groll M Bajorek M Köhler A Moroder L Rubin DM Huber R Glickman MH Finley D (2000) A gated channel into the proteasome core particle. Nat Struct Biol 7:1062–1067. 11062564 10.1038/80992

[B100] Groll M Ditzel L Löwe J Stock D Bochtler M Bartunik HD Huber R (1997) Structure of 20S proteasome from yeast at 2.4 A resolution. Nature 386:463–471. 9087403 10.1038/386463a0

[B101] Groll M Huber R (2003) Substrate access and processing by the 20S proteasome core particle. Int J Biochem Cell Biol 35:606–616. 12672453 10.1016/s1357-2725(02)00390-4

[B102] Groll M Huber R (2004) Inhibitors of the eukaryotic 20S proteasome core particle: a structural approach. Biochim Biophys Acta 1695:33–44. 15571807 10.1016/j.bbamcr.2004.09.025

[B103] Groothuis TA Reits EA (2005) Monitoring the distribution and dynamics of proteasomes in living cells. Methods Enzymol 399:549–563. 16338381 10.1016/S0076-6879(05)99037-X

[B104] Guerrero C Tagwerker C Kaiser P Huang L (2006) An integrated mass spectrometry-based proteomic approach: quantitative analysis of tandem affinity-purified in vivo cross-linked protein complexes (QTAX) to decipher the 26 S proteasome-interacting network. Mol Cell Proteomics 5:366–378. 16284124 10.1074/mcp.M500303-MCP200

[B105] Guerrero-Muñoz MJ Castillo-Carranza DL Kayed R (2014) Therapeutic approaches against common structural features of toxic oligomers shared by multiple amyloidogenic proteins. Biochem Pharmacol 88:468–478. 24406245 10.1016/j.bcp.2013.12.023

[B106] Guo Q Lehmer C Martínez-Sánchez A Rudack T Beck F Hartmann H Pérez-Berlanga M Frottin F Hipp MS Hartl FU (2018) In situ structure of neuronal C9orf72 poly-GA aggregates reveals proteasome recruitment. Cell 172:696–705.e12. 29398115 10.1016/j.cell.2017.12.030 PMC6035389

[B107] Haass C Selkoe DJ (2007) Soluble protein oligomers in neurodegeneration: lessons from the Alzheimer’s amyloid β-peptide. Nat Rev Mol Cell Biol 8:101–112. 17245412 10.1038/nrm2101

[B108] Hamer G Matilainen O Holmberg CI (2010) A photoconvertible reporter of the ubiquitin-proteasome system in vivo. Nat Methods 7:473–478. 20453865 10.1038/nmeth.1460

[B109] Hao M Zhang L An G Meng H Han Y Xie Z Xu Y Li C Yu Z Chang H (2011) Bone marrow stromal cells protect myeloma cells from bortezomib induced apoptosis by suppressing microRNA-15a expression. Leuk Lymphoma 52:1787–1794. 21534877 10.3109/10428194.2011.576791

[B110] Henderson A Erales J Hoyt MA Coffino P (2011) Dependence of proteasome processing rate on substrate unfolding. J Biol Chem 286:17495–17502. 21454622 10.1074/jbc.M110.212027 PMC3093823

[B111] Hershko A Ciechanover A (1998) The ubiquitin system. Annu Rev Biochem 67:425–479. 9759494 10.1146/annurev.biochem.67.1.425

[B112] Hershko A Ciechanover A Heller H Haas AL Rose IA (1980) Proposed role of ATP in protein breakdown: conjugation of protein with multiple chains of the polypeptide of ATP-dependent proteolysis. Proc Natl Acad Sci USA 77:1783–1786. 6990414 10.1073/pnas.77.4.1783 PMC348591

[B113] Hershko A Eytan E Ciechanover A Haas AL (1982) Immunochemical analysis of the turnover of ubiquitin-protein conjugates in intact cells. Relationship to the breakdown of abnormal proteins. J Biol Chem 257:13964–13970. 6292216

[B114] Hershko A Heller H Elias S Ciechanover A (1983) Components of ubiquitin-protein ligase system. Resolution, affinity purification, and role in protein breakdown. J Biol Chem 258:8206–8214. 6305978

[B115] Hewings DS Flygare JA Wertz IE Bogyo M (2017) Activity-based probes for the multicatalytic proteasome. FEBS J 284:1540–1554. 28107776 10.1111/febs.14016

[B116] Hideshima T Chauhan D Ishitsuka K Yasui H Raje N Kumar S Podar K Mitsiades C Hideshima H Bonham L (2005) Molecular characterization of PS-341 (bortezomib) resistance: implications for overcoming resistance using lysophosphatidic acid acyltransferase (LPAAT)-beta inhibitors. Oncogene 24:3121–3129. 15735676 10.1038/sj.onc.1208522

[B117] Hideshima T Chauhan D Richardson P Mitsiades C Mitsiades N Hayashi T Munshi N Dang L Castro A Palombella V (2002) NF-kappa B as a therapeutic target in multiple myeloma. J Biol Chem 277:16639–16647. 11872748 10.1074/jbc.M200360200

[B268] Holbeck SL Camalier R Crowell J Govindharajulu JP Hollingshead M Anderson LW Polley E Rubinstein L Srivastava A Wilsker D (2017) The National Cancer Institute ALMANAC: A comprehensive screening resource for the detection of anticancer drug pairs with enhanced therapeutic activity. Cancer Res. 77:3564–3576. 28446463 10.1158/0008-5472.CAN-17-0489 PMC5499996

[B118] Hough R Pratt G Rechsteiner M (1986) Ubiquitin-lysozyme conjugates. Identification and characterization of an ATP-dependent protease from rabbit reticulocyte lysates. J Biol Chem 261:2400–2408. 3003114

[B119] Hough R Pratt G Rechsteiner M (1987) Purification of two high molecular weight proteases from rabbit reticulocyte lysate. J Biol Chem 262:8303–8313. 3298229

[B120] Hoyt MA Zhang M Coffino P (2003) Ubiquitin-independent mechanisms of mouse ornithine decarboxylase degradation are conserved between mammalian and fungal cells. J Biol Chem 278:12135–12143. 12562772 10.1074/jbc.M211802200

[B121] Husnjak K Elsasser S Zhang N Chen X Randles L Shi Y Hofmann K Walters KJ Finley D Dikic I (2008) Proteasome subunit Rpn13 is a novel ubiquitin receptor. Nature 453:481–488. 18497817 10.1038/nature06926 PMC2839886

[B122] Huyer G Piluek WF Fansler Z Kreft SG Hochstrasser M Brodsky JL Michaelis S (2004) Distinct machinery is required in Saccharomyces cerevisiae for the endoplasmic reticulum-associated degradation of a multispanning membrane protein and a soluble luminal protein. J Biol Chem 279:38369–38378. 15252059 10.1074/jbc.M402468200

[B123] Ichikawa HT Conley T Muchamuel T Jiang J Lee S Owen T Barnard J Nevarez S Goldman BI Kirk CJ (2012) Beneficial effect of novel proteasome inhibitors in murine lupus via dual inhibition of type I interferon and autoantibody-secreting cells. Arthritis Rheum 64:493–503. 21905015 10.1002/art.33333 PMC4584406

[B124] Jarosch E Taxis C Volkwein C Bordallo J Finley D Wolf DH Sommer T (2002) Protein dislocation from the ER requires polyubiquitination and the AAA-ATPase Cdc48. Nat Cell Biol 4:134–139. 11813000 10.1038/ncb746

[B125] Johnson HWB Anderl JL Bradley EK Bui J Jones J Arastu-Kapur S Kelly LM Lowe E Moebius DC Muchamuel T (2017) Discovery of highly selective inhibitors of the immunoproteasome low molecular mass polypeptide 2 (LMP2) subunit. ACS Med Chem Lett 8:413–417. 28435528 10.1021/acsmedchemlett.6b00496 PMC5392757

[B126] Johnston SC Whitby FG Realini C Rechsteiner M Hill CP (1997) The proteasome 11S regulator subunit REG alpha (PA28 alpha) is a heptamer. Protein Sci 6:2469–2473. 9385652 10.1002/pro.5560061123 PMC2143584

[B127] Kayed R Head E Thompson JL McIntire TM Milton SC Cotman CW Glabe CG (2003) Common structure of soluble amyloid oligomers implies common mechanism of pathogenesis. Science 300:486–489. 12702875 10.1126/science.1079469

[B128] Keller JN Hanni KB Markesbery WR (2000) Impaired proteasome function in Alzheimer’s disease. J Neurochem 75:436–439. 10854289 10.1046/j.1471-4159.2000.0750436.x

[B129] Kerckhove N Collin A Condé S Chaleteix C Pezet D Balayssac D (2017) Long-term effects, pathophysiological mechanisms, and risk factors of chemotherapy-induced peripheral neuropathies: a comprehensive literature review. Front Pharmacol 8:86. 28286483 10.3389/fphar.2017.00086 PMC5323411

[B130] Khmelinskii A Blaszczak E Pantazopoulou M Fischer B Omnus DJ Le Dez G Brossard A Gunnarsson A Barry JD Meurer M (2014) Protein quality control at the inner nuclear membrane. Nature 516:410–413. 25519137 10.1038/nature14096 PMC4493439

[B131] Khor B Bredemeyer AL Huang C-Y Turnbull IR Evans R Maggi LB Jr White JM Walker LM Carnes K Hess RA (2006) Proteasome activator PA200 is required for normal spermatogenesis. Mol Cell Biol 26:2999–3007. 16581775 10.1128/MCB.26.8.2999-3007.2006 PMC1446934

[B132] Kim HT Goldberg AL (2017) The deubiquitinating enzyme Usp14 allosterically inhibits multiple proteasomal activities and ubiquitin-independent proteolysis. J Biol Chem 292:9830–9839. 28416611 10.1074/jbc.M116.763128 PMC5465503

[B133] King RW Deshaies RJ Peters JM Kirschner MW (1996) How proteolysis drives the cell cycle. Science 274:1652–1659. 8939846 10.1126/science.274.5293.1652

[B134] Kisselev AF Callard A Goldberg AL (2006) Importance of the different proteolytic sites of the proteasome and the efficacy of inhibitors varies with the protein substrate. J Biol Chem 281:8582–8590. 16455650 10.1074/jbc.M509043200

[B135] Kisselev AF Goldberg AL (2001) Proteasome inhibitors: from research tools to drug candidates. Chem Biol 8:739–758. 11514224 10.1016/s1074-5521(01)00056-4

[B136] Kisselev AF Kaganovich D Goldberg AL (2002) Binding of hydrophobic peptides to several non-catalytic sites promotes peptide hydrolysis by all active sites of 20 S proteasomes. Evidence for peptide-induced channel opening in the α-rings. J Biol Chem 277:22260–22270. 11927581 10.1074/jbc.M112360200

[B137] Kisselev AF van der Linden WA Overkleeft HS (2012) Proteasome inhibitors: an expanding army attacking a unique target. Chem Biol 19:99–115. 22284358 10.1016/j.chembiol.2012.01.003 PMC3503453

[B138] Kniepert A Groettrup M (2014) The unique functions of tissue-specific proteasomes. Trends Biochem Sci 39:17–24. 24286712 10.1016/j.tibs.2013.10.004

[B139] Knowles SE Ballard FJ (1976) Selective control of the degradation of normal and aberrant proteins in Reuber H35 hepatoma cells. Biochem J 156:609–617. 182157 10.1042/bj1560609 PMC1163795

[B140] Knowles SE Gunn JM Hanson RW Ballard FJ (1975) Increased degradation rates of protein synthesized in hepatoma cells in the presence of amino acid analogues. Biochem J 146:595–600. 167723 10.1042/bj1460595 PMC1165348

[B141] Köhler A Cascio P Leggett DS Woo KM Goldberg AL Finley D (2001) The axial channel of the proteasome core particle is gated by the Rpt2 ATPase and controls both substrate entry and product release. Mol Cell 7:1143–1152. 11430818 10.1016/s1097-2765(01)00274-x

[B142] Kraus M Bader J Geurink PP Weyburne ES Mirabella AC Silzle T Shabaneh TB van der Linden WA de Bruin G Haile SR (2015) The novel β2-selective proteasome inhibitor LU-102 synergizes with bortezomib and carfilzomib to overcome proteasome inhibitor resistance of myeloma cells. Haematologica 100:1350–1360. 26069288 10.3324/haematol.2014.109421 PMC4591768

[B266] Kuhn DJ Chen Q Voorhees PM Strader JS Shenk KD Sun CM Demo SD Bennett MK van Leeuwen FW Chanan-Khan AA (2007) Potent activity of carfilzomib, a novel, irreversible inhibitor of the ubiquitin-proteasome pathway, against preclinical models of multiple myeloma. Blood. 110:3281–3290. 17591945 10.1182/blood-2007-01-065888 PMC2200918

[B143] Kuhn DJ Orlowski RZ Bjorklund CC (2011) Second generation proteasome inhibitors: carfilzomib and immunoproteasome-specific inhibitors (IPSIs). Curr Cancer Drug Targets 11:285–295. 21247387 10.2174/156800911794519725

[B144] Kuo CL Collins GA Goldberg AL (2018) Methods to rapidly prepare mammalian 26S proteasomes for biochemical analysis. Methods Mol Biol 1844:277–288. 30242716 10.1007/978-1-4939-8706-1_18 PMC6427908

[B267] Kupperman E Lee EC Cao Y Bannerman B Fitzgerald M Berger A Yu J Yang Y Hales P Bruzzese F (2010) Evaluation of the proteasome inhibitor MLN9708 in preclinical models of human cancer. Cancer Res. 70:1970–1980. 20160034 10.1158/0008-5472.CAN-09-2766

[B145] Laubach JP Mitsiades CS Roccaro AM Ghobrial IM Anderson KC Richardson PG (2009) Clinical challenges associated with bortezomib therapy in multiple myeloma and Waldenström’s macroglobulinemia. Leuk Lymphoma 50:694–702. 19452315 10.1080/10428190902866732 PMC3133638

[B146] Layfield R Lowe J Bedford L (2005) The ubiquitin-proteasome system and neurodegenerative disorders. Essays Biochem 41:157–171. 16250904 10.1042/EB0410157

[B147] Lecker SH Goldberg AL Mitch WE (2006) Protein degradation by the ubiquitin-proteasome pathway in normal and disease states. J Am Soc Nephrol 17:1807–1819. 16738015 10.1681/ASN.2006010083

[B148] Lee B-HH Lee MJ Park S Oh D-CC Elsasser S Chen PC Gartner C Dimova N Hanna J Gygi SP (2010) Enhancement of proteasome activity by a small-molecule inhibitor of USP14. Nature 467:179–184. 20829789 10.1038/nature09299 PMC2939003

[B149] Leggett DS Hanna J Borodovsky A Crosas B Schmidt M Baker RT Walz T Ploegh H Finley D (2002) Multiple associated proteins regulate proteasome structure and function. Mol Cell 10:495–507. 12408819 10.1016/s1097-2765(02)00638-x

[B150] Levin N Spencer A Harrison SJ Chauhan D Burrows FJ Anderson KC Reich SD Richardson PG Trikha M (2016) Marizomib irreversibly inhibits proteasome to overcome compensatory hyperactivation in multiple myeloma and solid tumour patients. Br J Haematol 174:711–720. 27161872 10.1111/bjh.14113 PMC5084787

[B151] Li H Chen Z Hu T Wang L Yu Y Zhao Y Sun W Guan S Pang JC Woodfield SE (2016) Novel proteasome inhibitor ixazomib sensitizes neuroblastoma cells to doxorubicin treatment. Sci Rep 6:34397. 27687684 10.1038/srep34397 PMC5043366

[B152] Li J Yakushi T Parlati F Mackinnon AL Perez C Ma Y Carter KP Colayco S Magnuson G Brown B (2017) Capzimin is a potent and specific inhibitor of proteasome isopeptidase Rpn11. Nat Chem Biol 13:486–493. 28244987 10.1038/nchembio.2326 PMC5570473

[B153] Li X Wang CE Huang S Xu X Li XJ Li H Li S (2010) Inhibiting the ubiquitin-proteasome system leads to preferential accumulation of toxic N-terminal mutant huntingtin fragments. Hum Mol Genet 19:2445–2455. 20354076 10.1093/hmg/ddq127 PMC2876889

[B270] Li X Zhao X Fang Y Jiang X Duong T Fan C Huang CC Kain SR (1998) Generation of destabilized green fluorescent protein as a transcription reporter. J Biol Chem. 273:34970–34975. 9857028 10.1074/jbc.273.52.34970

[B154] Lickliter J, Anderl J, Kirk CJ, Wang J, and Bomba D (2017) KZR-616, a selective inhibitor of the immunoproteasome, shows a promising safety and target inhibition profile in a phase I, double-blind, single (SAD) and multiple ascending dose (MAD) study in healthy volunteers (Abstract). *Arthritis Rheumatol* 69 (Suppl 10):2587.

[B155] Lindersson E Beedholm R Højrup P Moos T Gai W Hendil KB Jensen PH (2004) Proteasomal inhibition by α-synuclein filaments and oligomers. J Biol Chem 279:12924–12934. 14711827 10.1074/jbc.M306390200

[B156] Lindsten K Menéndez-Benito V Masucci MG Dantuma NP (2003) A transgenic mouse model of the ubiquitin/proteasome system. Nat Biotechnol 21:897–902. 12872133 10.1038/nbt851

[B157] Liu H Wan C Ding Y Han R He Y Xiao J Hao J (2017) PR-957, a selective inhibitor of immunoproteasome subunit low-MW polypeptide 7, attenuates experimental autoimmune neuritis by suppressing T_h_17-cell differentiation and regulating cytokine production. FASEB J 31:1756–1766. 28096232 10.1096/fj.201601147R

[B158] Lü S Chen Z Yang J Chen L Gong S Zhou H Guo L Wang J (2008) Overexpression of the PSMB5 gene contributes to bortezomib resistance in T-lymphoblastic lymphoma/leukemia cells derived from Jurkat line. Exp Hematol 36:1278–1284. 18562081 10.1016/j.exphem.2008.04.013

[B159] Luker GD Pica CM Song J Luker KE Piwnica-Worms D (2003) Imaging 26S proteasome activity and inhibition in living mice. Nat Med 9:969–973. 12819780 10.1038/nm894

[B160] Machiels BM Henfling ME Gerards WL Broers JL Bloemendal H Ramaekers FC Schutte B (1997) Detailed analysis of cell cycle kinetics upon proteasome inhibition. Cytometry 28:243–252. 9222110

[B161] Martinez-Fonts K Matouschek A (2016) A rapid and versatile method for generating proteins with defined ubiquitin chains. Biochemistry 55:1898–1908. 26943792 10.1021/acs.biochem.5b01310 PMC4922136

[B162] Masdehors P Omura S Merle-Béral H Mentz F Cosset JM Dumont J Magdelénat H Delic J (1999) Increased sensitivity of CLL-derived lymphocytes to apoptotic death activation by the proteasome-specific inhibitor lactacystin. Br J Haematol 105:752–757. 10354141 10.1046/j.1365-2141.1999.01388.x

[B163] Mateos M-V Masszi T Grzasko N Hansson M Sandhu I Pour L Viterbo L Jackson SR Stoppa A-M Gimsing P (2017) Impact of prior therapy on the efficacy and safety of oral ixazomib-lenalidomide-dexamethasone vs. placebo-lenalidomide-dexamethasone in patients with relapsed/refractory multiple myeloma in TOURMALINE-MM1. Haematologica 102:1767–1775. 28751562 10.3324/haematol.2017.170118 PMC5622861

[B164] Matyskiela ME Lander GC Martin A (2013) Conformational switching of the 26S proteasome enables substrate degradation. Nat Struct Mol Biol 20:781–788. 23770819 10.1038/nsmb.2616 PMC3712289

[B165] McKinnon C Tabrizi SJ (2014) The ubiquitin-proteasome system in neurodegeneration. Antioxid Redox Signal 21:2302–2321. 24437518 10.1089/ars.2013.5802

[B166] McNaught KS Mytilineou C Jnobaptiste R Yabut J Shashidharan P Jennert P Olanow CW (2002) Impairment of the ubiquitin-proteasome system causes dopaminergic cell death and inclusion body formation in ventral mesencephalic cultures. J Neurochem 81:301–306. 12064477 10.1046/j.1471-4159.2002.00821.x

[B167] McNaught KS Olanow CW Halliwell B Isacson O Jenner P (2001) Failure of the ubiquitin-proteasome system in Parkinson’s disease. Nat Rev Neurosci 2:589–594. 11484002 10.1038/35086067

[B168] McNaught KS Perl DP Brownell AL Olanow CW (2004) Systemic exposure to proteasome inhibitors causes a progressive model of Parkinson’s disease. Ann Neurol 56:149–162. 15236415 10.1002/ana.20186

[B169] Menéndez-Benito V Heessen S Dantuma NP (2005) Monitoring of ubiquitin-dependent proteolysis with green fluorescent protein substrates. Methods Enzymol 399:490–511. 16338378 10.1016/S0076-6879(05)99034-4

[B170] Moreau P Masszi T Grzasko N Bahlis NJ Hansson M Pour L Sandhu I Ganly P Baker BW Jackson SR TOURMALINE-MM1 Study Group (2016) Oral ixazomib, lenalidomide, and dexamethasone for multiple myeloma. N Engl J Med 374:1621–1634. 27119237 10.1056/NEJMoa1516282

[B171] Morris EP da Fonseca PCA (2017) High-resolution cryo-EM proteasome structures in drug development. Acta Crystallogr D Struct Biol 73:522–533. 28580914 10.1107/S2059798317007021 PMC5458494

[B172] Muchamuel T Basler M Aujay MA Suzuki E Kalim KW Lauer C Sylvain C Ring ER Shields J Jiang J (2009) A selective inhibitor of the immunoproteasome subunit LMP7 blocks cytokine production and attenuates progression of experimental arthritis. Nat Med 15:781–787. 19525961 10.1038/nm.1978

[B173] Murakami Y Matsufuji S Kameji T Hayashi S Igarashi K Tamura T Tanaka K Ichihara A (1992) Ornithine decarboxylase is degraded by the 26S proteasome without ubiquitination. Nature 360:597–599. 1334232 10.1038/360597a0

[B174] Murata S Sasaki K Kishimoto T Niwa S Hayashi H Takahama Y Tanaka K (2007) Regulation of CD8+ T cell development by thymus-specific proteasomes. Science 316:1349–1353. 17540904 10.1126/science.1141915

[B175] Muz B Ghazarian RN Ou M Luderer MJ Kusdono HD Azab AK (2016) Spotlight on ixazomib: potential in the treatment of multiple myeloma. Drug Des Devel Ther 10:217–226. 10.2147/DDDT.S93602 PMC4714737 26811670

[B176] Oerlemans R Franke NE Assaraf YG Cloos J van Zantwijk I Berkers CR Scheffer GL Debipersad K Vojtekova K Lemos C (2008) Molecular basis of bortezomib resistance: proteasome subunit β5 (PSMB5) gene mutation and overexpression of PSMB5 protein. Blood 112:2489–2499. 18565852 10.1182/blood-2007-08-104950

[B177] Ortega J Heymann JB Kajava AV Ustrell V Rechsteiner M Steven AC (2005) The axial channel of the 20S proteasome opens upon binding of the PA200 activator. J Mol Biol 346:1221–1227. 15713476 10.1016/j.jmb.2004.12.049

[B178] Ortega Z Díaz-Hernández M Lucas JJ (2007) Is the ubiquitin-proteasome system impaired in Huntington’s disease? Cell Mol Life Sci 64:2245–2257. 17604996 10.1007/s00018-007-7222-8 PMC11136357

[B179] Otero MG Alloatti M Cromberg LE Almenar-Queralt A Encalada SE Pozo Devoto VM Bruno L Goldstein LSB Falzone TL (2014) Fast axonal transport of the proteasome complex depends on membrane interaction and molecular motor function. J Cell Sci 127:1537–1549. 24522182 10.1242/jcs.140780

[B180] Pagano M Tam SW Theodoras AM Beer-Romero P Del Sal G Chau V Yew PR Draetta GF Rolfe M (1995) Role of the ubiquitin-proteasome pathway in regulating abundance of the cyclin-dependent kinase inhibitor p27. Science 269:682–685. 7624798 10.1126/science.7624798

[B181] Palombella VJ Rando OJ Goldberg AL Maniatis T (1994) The ubiquitin-proteasome pathway is required for processing the NF-kappa B1 precursor protein and the activation of NF-kappa B. Cell 78:773–785. 8087845 10.1016/s0092-8674(94)90482-0

[B182] Peters JM (2002) The anaphase-promoting complex: proteolysis in mitosis and beyond. Mol Cell 9:931–943. 12049731 10.1016/s1097-2765(02)00540-3

[B183] Peth A Besche HC Goldberg AL (2009) Ubiquitinated proteins activate the proteasome by binding to Usp14/Ubp6, which causes 20S gate opening. Mol Cell 36:794–804. 20005843 10.1016/j.molcel.2009.11.015 PMC2796264

[B184] Peth A Kukushkin N Bossé M Goldberg AL (2013) Ubiquitinated proteins activate the proteasomal ATPases by binding to Usp14 or Uch37 homologs. J Biol Chem 288:7781–7790. 23341450 10.1074/jbc.M112.441907 PMC3597817

[B185] Peth A Uchiki T Goldberg AL (2010) ATP-dependent steps in the binding of ubiquitin conjugates to the 26S proteasome that commit to degradation. Mol Cell 40:671–681. 21095592 10.1016/j.molcel.2010.11.002 PMC3038635

[B186] Pickering AM Davies KJA (2012) Differential roles of proteasome and immunoproteasome regulators Pa28αβ, Pa28γ and Pa200 in the degradation of oxidized proteins. Arch Biochem Biophys 523:181–190. 22564544 10.1016/j.abb.2012.04.018 PMC3384713

[B188] Prakash S Tian L Ratliff KS Lehotzky RE Matouschek A (2004) An unstructured initiation site is required for efficient proteasome-mediated degradation. Nat Struct Mol Biol 11:830–837. 15311270 10.1038/nsmb814

[B189] Qian MX Pang Y Liu CH Haratake K Du BY Ji DY Wang GF Zhu QQ Song W Yu Y (2013) Acetylation-mediated proteasomal degradation of core histones during DNA repair and spermatogenesis. Cell 153:1012–1024. 23706739 10.1016/j.cell.2013.04.032 PMC3983474

[B190] Qiu XB Ouyang SY Li CJ Miao S Wang L Goldberg AL (2006) hRpn13/ADRM1/GP110 is a novel proteasome subunit that binds the deubiquitinating enzyme, UCH37. EMBO J 25:5742–5753. 17139257 10.1038/sj.emboj.7601450 PMC1698896

[B191] Rabinovich E Kerem A Fröhlich K-U Diamant N Bar-Nun S (2002) AAA-ATPase p97/Cdc48p, a cytosolic chaperone required for endoplasmic reticulum-associated protein degradation. Mol Cell Biol 22:626–634. 11756557 10.1128/MCB.22.2.626-634.2002 PMC139744

[B192] Rabl J Smith DM Yu Y Chang SC Goldberg AL Cheng Y (2008) Mechanism of gate opening in the 20S proteasome by the proteasomal ATPases. Mol Cell 30:360–368. 18471981 10.1016/j.molcel.2008.03.004 PMC4141531

[B193] Ramachandran KV Margolis SS (2017) A mammalian nervous-system-specific plasma membrane proteasome complex that modulates neuronal function. Nat Struct Mol Biol 24:419–430. 28287632 10.1038/nsmb.3389 PMC5383508

[B194] Rechsteiner M Hill CP (2005) Mobilizing the proteolytic machine: cell biological roles of proteasome activators and inhibitors. Trends Cell Biol 15:27–33. 15653075 10.1016/j.tcb.2004.11.003

[B195] Reits E Griekspoor A Neijssen J Groothuis T Jalink K van Veelen P Janssen H Calafat J Drijfhout JW Neefjes J (2003) Peptide diffusion, protection, and degradation in nuclear and cytoplasmic compartments before antigen presentation by MHC class I. Immunity 18:97–108. 12530979 10.1016/s1074-7613(02)00511-3

[B196] Religa TL Sprangers R Kay LE (2010) Dynamic regulation of archaeal proteasome gate opening as studied by TROSY NMR. Science 328:98–102. 20360109 10.1126/science.1184991

[B197] Roccaro AM Sacco A Aujay M Ngo HT Azab AK Azab F Quang P Maiso P Runnels J Anderson KC (2010) Selective inhibition of chymotrypsin-like activity of the immunoproteasome and constitutive proteasome in Waldenstrom macroglobulinemia. Blood 115:4051–4060. 20110419 10.1182/blood-2009-09-243402 PMC2875093

[B198] Rock KL Goldberg AL (1999) Degradation of cell proteins and the generation of MHC class I-presented peptides. Annu Rev Immunol 17:739–779. 10358773 10.1146/annurev.immunol.17.1.739

[B199] Rock KL Gramm C Rothstein L Clark K Stein R Dick L Hwang D Goldberg AL (1994) Inhibitors of the proteasome block the degradation of most cell proteins and the generation of peptides presented on MHC class I molecules. Cell 78:761–771. 8087844 10.1016/s0092-8674(94)90462-6

[B200] Roelofs J Suppahia A Waite KA Park S (2018) Native gel approaches in studying proteasome assembly and chaperones. Methods Mol Biol 1844:237–260. 30242714 10.1007/978-1-4939-8706-1_16 PMC6743976

[B201] Rubinsztein DC (2006) The roles of intracellular protein-degradation pathways in neurodegeneration. Nature 443:780–786. 17051204 10.1038/nature05291

[B202] Ruschak AM Kay LE (2012) Proteasome allostery as a population shift between interchanging conformers. Proc Natl Acad Sci USA 109:E3454–E3462. 23150576 10.1073/pnas.1213640109 PMC3528551

[B203] Sadre-Bazzaz K Whitby FG Robinson H Formosa T Hill CP (2010) Structure of a Blm10 complex reveals common mechanisms for proteasome binding and gate opening. Mol Cell 37:728–735. 20227375 10.1016/j.molcel.2010.02.002 PMC2859072

[B204] Sakamoto KM (2002) Ubiquitin-dependent proteolysis: its role in human diseases and the design of therapeutic strategies. Mol Genet Metab 77:44–56. 12359129 10.1016/s1096-7192(02)00146-4

[B271] Santos RLA Bai L Singh PK Murakami N Fan H Zhan W Zhu Y Jiang X Zhang K Assker JP (2017) Structure of human immunoproteasome with a reversible and noncompetitive inhibitor that selectively inhibits activated lymphocytes. Nat Commun 8:1692. 10.1038/s41467-017-01760-5 PMC5700161 29167449

[B205] Scherer DC Brockman JA Chen Z Maniatis T Ballard DW (1995) Signal-induced degradation of I kappa B alpha requires site-specific ubiquitination. Proc Natl Acad Sci USA 92:11259–11263. 7479976 10.1073/pnas.92.24.11259 PMC40611

[B206] Schimke RT (1964) The importance of both synthesis and degradation in the control of arginase levels in rat liver. J Biol Chem 239:3808–3817. 14257612

[B207] Schimke RT Doyle D (1970) Control of enzyme levels in animal tissues. Annu Rev Biochem 39:929–976. 4394639 10.1146/annurev.bi.39.070170.004433

[B208] Schlafer D Shah KS Panjic EH Lonial S (2017) Safety of proteasome inhibitors for treatment of multiple myeloma. Expert Opin Drug Saf 16:167–183. 27841029 10.1080/14740338.2017.1259310

[B209] Schmidt M Finley D (2014) Regulation of proteasome activity in health and disease. Biochim Biophys Acta 1843:13–25. 23994620 10.1016/j.bbamcr.2013.08.012 PMC3858528

[B210] Schmidt M Haas W Crosas B Santamaria PG Gygi SP Walz T Finley D (2005) The HEAT repeat protein Blm10 regulates the yeast proteasome by capping the core particle. Nat Struct Mol Biol 12:294–303. 15778719 10.1038/nsmb914

[B211] Schoenheimer R (1942) The Dynamic State of Body Constituents, Harvard University Press, Cambridge, MA.

[B212] Schoenheimer R Ratner S Rittenberg D (1939) Studies in protein metabolism: VII. The metabolism of tyrosine. J Biol Chem 127:333–344.

[B213] Screen M Britton M Downey SL Verdoes M Voges MJ Blom AEM Geurink PP Risseeuw MDP Florea BI van der Linden WA (2010) Nature of pharmacophore influences active site specificity of proteasome inhibitors. J Biol Chem 285:40125–40134. 20937826 10.1074/jbc.M110.160606 PMC3000995

[B214] Selkoe DJ (2003) Folding proteins in fatal ways. Nature 426:900–904. 14685251 10.1038/nature02264

[B215] Sha Z Zhao J Goldberg AL (2018) Measuring the overall rate of protein breakdown in cells and the contributions of the ubiquitin-proteasome and autophagy-lysosomal pathways. Methods Mol Biol 1844:261–276. 30242715 10.1007/978-1-4939-8706-1_17 PMC6441977

[B216] Shah SA Potter MW McDade TP Ricciardi R Perugini RA Elliott PJ Adams J Callery MP (2001) 26S proteasome inhibition induces apoptosis and limits growth of human pancreatic cancer. J Cell Biochem 82:110–122. 11400168 10.1002/jcb.1150

[B217] Simpson MV (1953) The release of labeled amino acids from the proteins of rat liver slices. J Biol Chem 201:143–154. 13044783

[B218] Singh Gautam AK Martinez-Fonts K Matouschek A (2018) Scalable in vitro proteasome activity assay. Methods Mol Biol 1844:321–341. 30242719 10.1007/978-1-4939-8706-1_21

[B219] Śledź P Unverdorben P Beck F Pfeifer G Schweitzer A Förster F Baumeister W (2013) Structure of the 26S proteasome with ATP-γS bound provides insights into the mechanism of nucleotide-dependent substrate translocation. Proc Natl Acad Sci USA 110:7264–7269. 23589842 10.1073/pnas.1305782110 PMC3645540

[B220] Smith AJ Dai H Correia C Takahashi R Lee SH Schmitz I Kaufmann SH (2011) Noxa/Bcl-2 protein interactions contribute to bortezomib resistance in human lymphoid cells. J Biol Chem 286:17682–17692. 21454712 10.1074/jbc.M110.189092 PMC3093844

[B221] Smith DM Benaroudj N Goldberg A (2006) Proteasomes and their associated ATPases: a destructive combination. J Struct Biol 156:72–83. 16919475 10.1016/j.jsb.2006.04.012

[B222] Smith DM Chang SC Park S Finley D Cheng Y Goldberg AL (2007) Docking of the proteasomal ATPases’ carboxyl termini in the 20S proteasome’s α ring opens the gate for substrate entry. Mol Cell 27:731–744. 17803938 10.1016/j.molcel.2007.06.033 PMC2083707

[B223] Smith DM Kafri G Cheng Y Ng D Walz T Goldberg AL (2005) ATP binding to PAN or the 26S ATPases causes association with the 20S proteasome, gate opening, and translocation of unfolded proteins. Mol Cell 20:687–698. 16337593 10.1016/j.molcel.2005.10.019

[B224] Snoberger A Anderson RT Smith DM (2017) The proteasomal ATPases use a slow but highly processive strategy to unfold proteins. Front Mol Biosci 4:18. 28421184 10.3389/fmolb.2017.00018 PMC5378721

[B225] Spencer E Jiang J Chen ZJ (1999) Signal-induced ubiquitination of IkappaBalpha by the F-box protein Slimb/beta-TrCP. Genes Dev 13:284–294. 9990853 10.1101/gad.13.3.284 PMC316434

[B226] Staff NP Podratz JL Grassner L Bader M Paz J Knight AM Loprinzi CL Trushina E Windebank AJ (2013) Bortezomib alters microtubule polymerization and axonal transport in rat dorsal root ganglion neurons. Neurotoxicology 39:124–131. 24035926 10.1016/j.neuro.2013.09.001 PMC3844018

[B227] Steele JM (2013) Carfilzomib: a new proteasome inhibitor for relapsed or refractory multiple myeloma. J Oncol Pharm Pract 19:348–354. 23292972 10.1177/1078155212470388

[B228] Sugumar D Keller J Vij R (2015) Targeted treatments for multiple myeloma: specific role of carfilzomib. Pharm Genomics Pers Med 8:23–33. 10.2147/PGPM.S39085 PMC4325627 25691814

[B229] Tai H-C Schuman EM (2008) Ubiquitin, the proteasome and protein degradation in neuronal function and dysfunction. Nat Rev Neurosci 9:826–838. 18931696 10.1038/nrn2499

[B230] Tanaka K (1994) Role of proteasomes modified by interferon-γ in antigen processing. J Leukoc Biol 56:571–575. 7964165 10.1002/jlb.56.5.571

[B231] Tanaka K Waxman L Goldberg AL (1983) ATP serves two distinct roles in protein degradation in reticulocytes, one requiring and one independent of ubiquitin. J Cell Biol 96:1580–1585. 6304111 10.1083/jcb.96.6.1580 PMC2112434

[B232] Tanaka K Yoshimura T Kumatori A Ichihara A Ikai A Nishigai M Kameyama K Takagi T (1988) Proteasomes (multi-protease complexes) as 20 S ring-shaped particles in a variety of eukaryotic cells. J Biol Chem 263:16209–16217. 3141402

[B233] Thibaudeau TA Anderson RT Smith DM (2018) A common mechanism of proteasome impairment by neurodegenerative disease-associated oligomers. Nat Commun 9:1097. 29545515 10.1038/s41467-018-03509-0 PMC5854577

[B234] Thrower JS Hoffman L Rechsteiner M Pickart CM (2000) Recognition of the polyubiquitin proteolytic signal. EMBO J 19:94–102. 10619848 10.1093/emboj/19.1.94 PMC1171781

[B235] Travers KJ Patil CK Wodicka L Lockhart DJ Weissman JS Walter P (2000) Functional and genomic analyses reveal an essential coordination between the unfolded protein response and ER-associated degradation. Cell 101:249–258. 10847680 10.1016/s0092-8674(00)80835-1

[B236] Tsai YC Weissman AM (2010) The unfolded protein response, degradation from the endoplasmic reticulum and cancer. Genes Cancer 1:764–778. 21331300 10.1177/1947601910383011 PMC3039444

[B237] Tseng BP Green KN Chan JL Blurton-Jones M LaFerla FM (2008) Abeta inhibits the proteasome and enhances amyloid and tau accumulation. Neurobiol Aging 29:1607–1618. 17544172 10.1016/j.neurobiolaging.2007.04.014 PMC2664168

[B238] Unverdorben P Beck F Śledź P Schweitzer A Pfeifer G Plitzko JM Baumeister W Förster F (2014) Deep classification of a large cryo-EM dataset defines the conformational landscape of the 26S proteasome. Proc Natl Acad Sci USA 111:5544–5549. 24706844 10.1073/pnas.1403409111 PMC3992697

[B239] Ustrell V Hoffman L Pratt G Rechsteiner M (2002) PA200, a nuclear proteasome activator involved in DNA repair. EMBO J 21:3516–3525. 12093752 10.1093/emboj/cdf333 PMC126083

[B240] Van Kaer L Ashton-Rickardt PG Eichelberger M Gaczynska M Nagashima K Rock KL Goldberg AL Doherty PC Tonegawa S (1994) Altered peptidase and viral-specific T cell response in LMP2 mutant mice. Immunity 1:533–541. 7600282 10.1016/1074-7613(94)90043-4

[B241] van Tellingen O Yetkin-Arik B de Gooijer MC Wesseling P Wurdinger T de Vries HE (2015) Overcoming the blood-brain tumor barrier for effective glioblastoma treatment. Drug Resist Updat 19:1–12. 25791797 10.1016/j.drup.2015.02.002

[B242] Varshavsky A (2005) Ubiquitin fusion technique and related methods. Methods Enzymol 399:777–799. 16338395 10.1016/S0076-6879(05)99051-4

[B243] Varshavsky A Bachmair A Finley D (1987) The N-end rule of selective protein turnover: mechanistic aspects and functional implications. Biochem Soc Trans 15:815–816. 3691950 10.1042/bst0150815

[B244] Vashist S Ng DTW (2004) Misfolded proteins are sorted by a sequential checkpoint mechanism of ER quality control. J Cell Biol 165:41–52. 15078901 10.1083/jcb.200309132 PMC2172089

[B245] Verma R Aravind L Oania R McDonald WH Yates JR 3rd Koonin EV Deshaies RJ (2002) Role of Rpn11 metalloprotease in deubiquitination and degradation by the 26S proteasome. Science 298:611–615. 12183636 10.1126/science.1075898

[B246] Voges D Zwickl P Baumeister W (1999) The 26S proteasome: a molecular machine designed for controlled proteolysis. Annu Rev Biochem 68:1015–1068. 10872471 10.1146/annurev.biochem.68.1.1015

[B247] von Brzezinski L Säring P Landgraf P Cammann C Seifert U Dieterich DC (2017) Low neurotoxicity of ONX-0914 supports the idea of specific immunoproteasome inhibition as a side-effect-limiting, therapeutic strategy. Eur J Microbiol Immunol (Bp) 7:234–245. 29034113 10.1556/1886.2017.00025 PMC5632751

[B248] Wang CY Mayo MW Baldwin AS Jr (1996) TNF- and cancer therapy-induced apoptosis: potentiation by inhibition of NF-kappaB. Science 274:784–787. 8864119 10.1126/science.274.5288.784

[B249] Wang CY Mayo MW Korneluk RG Goeddel DV Baldwin AS Jr (1998) NF-kappaB antiapoptosis: induction of TRAF1 and TRAF2 and c-IAP1 and c-IAP2 to suppress caspase-8 activation. Science 281:1680–1683. 9733516 10.1126/science.281.5383.1680

[B250] Wang J Wang CE Orr A Tydlacka S Li SH Li XJ (2008) Impaired ubiquitin-proteasome system activity in the synapses of Huntington’s disease mice. J Cell Biol 180:1177–1189. 18362179 10.1083/jcb.200709080 PMC2290845

[B251] Wang X Cimermancic P Yu C Schweitzer A Chopra N Engel JL Greenberg C Huszagh AS Beck F Sakata E (2017) Molecular details underlying dynamic structures and regulation of the human 26S proteasome. Mol Cell Proteomics 16:840–854. 28292943 10.1074/mcp.M116.065326 PMC5417825

[B252] Wehmer M Rudack T Beck F Aufderheide A Pfeifer G Plitzko JM Förster F Schulten K Baumeister W Sakata E (2017) Structural insights into the functional cycle of the ATPase module of the 26S proteasome. Proc Natl Acad Sci USA 114:1305–1310. 28115689 10.1073/pnas.1621129114 PMC5307450

[B253] Wilk S Orlowski M (1980) Cation-sensitive neutral endopeptidase: isolation and specificity of the bovine pituitary enzyme. J Neurochem 35:1172–1182. 6778972 10.1111/j.1471-4159.1980.tb07873.x

[B254] Wilk S Orlowski M (1983) Evidence that pituitary cation-sensitive neutral endopeptidase is a multicatalytic protease complex. J Neurochem 40:842–849. 6338156 10.1111/j.1471-4159.1983.tb08056.x

[B255] Wilkinson KD Urban MK Haas AL (1980) Ubiquitin is the ATP-dependent proteolysis factor I of rabbit reticulocytes. J Biol Chem 255:7529–7532. 6249803

[B256] Winston JT Strack P Beer-Romero P Chu CY Elledge SJ Harper JW (1999) The SCFbeta-TRCP-ubiquitin ligase complex associates specifically with phosphorylated destruction motifs in IkappaBalpha and β-catenin and stimulates IkappaBalpha ubiquitination in vitro. Genes Dev 13:270–283. 9990852 10.1101/gad.13.3.270 PMC316433

[B257] Witkowska J Giżyńska M Grudnik P Golik P Karpowicz P Giełdoń A Dubin G Jankowska E (2017) Crystal structure of a low molecular weight activator Blm-pep with yeast 20S proteasome - insights into the enzyme activation mechanism. Sci Rep 7:6177. 28733623 10.1038/s41598-017-05997-4 PMC5522460

[B258] Wu X Rapoport TA (2018) Mechanistic insights into ER-associated protein degradation. Curr Opin Cell Biol 53:22–28. 29719269 10.1016/j.ceb.2018.04.004 PMC6131047

[B259] Wu Y Luo H Kanaan N Wu J (2000) The proteasome controls the expression of a proliferation-associated nuclear antigen Ki-67. J Cell Biochem 76:596–604. 10653979

[B260] Yu Y Smith DM Kim HM Rodriguez V Goldberg AL Cheng Y (2010) Interactions of PAN’s C-termini with archaeal 20S proteasome and implications for the eukaryotic proteasome-ATPase interactions. EMBO J 29:692–702. 20019667 10.1038/emboj.2009.382 PMC2830694

[B261] Zavortink M Thacher T Rechsteiner M (1979) Degradation of proteins microinjected into cultured mammalian cells. J Cell Physiol 100:175–185. 112104 10.1002/jcp.1041000118

[B272] Zhou HJ Aujay MA Bennett MK Dajee M Demo SD Fang Y Ho MN Jiang J Kirk CJ Laidig GJ (2009) Design and synthesis of an orally bioavailable and selective peptide epoxyketone proteasome inhibitor (PR-047). J Med Chem. 52:3028–3038. 19348473 10.1021/jm801329v

[B262] Zhu YX Tiedemann R Shi CX Yin H Schmidt JE Bruins LA Keats JJ Braggio E Sereduk C Mousses S (2011) RNAi screen of the druggable genome identifies modulators of proteasome inhibitor sensitivity in myeloma including CDK5. Blood 117:3847–3857. 21289309 10.1182/blood-2010-08-304022 PMC3083298

[B263] Zong WX Edelstein LC Chen C Bash J Gélinas C (1999) The prosurvival Bcl-2 homolog Bfl-1/A1 is a direct transcriptional target of NF-kappaB that blocks TNFalpha-induced apoptosis. Genes Dev 13:382–387. 10049353 10.1101/gad.13.4.382 PMC316475

